# Smartphone App Designed to Collect Health Information in Older Adults: Usability Study

**DOI:** 10.2196/56653

**Published:** 2024-05-30

**Authors:** Joanne M Murabito, Jamie M Faro, Yuankai Zhang, Angelo DeMalia, Alexander Hamel, Nakesha Agyapong, Hongshan Liu, Eric Schramm, David D McManus, Belinda Borrelli

**Affiliations:** 1 Section of General Internal Medicine Department of Medicine Boston University Chobanian & Avedisian School of Medicine Boston, MA United States; 2 Framingham Heart Study Boston University Framingham, MA United States; 3 Department of Medicine University of Massachusetts Chan Medical School Worcester, MA United States; 4 Department of Biostatistics Boston University School of Public Health Boston, MA United States; 5 CareEvolution, Inc Ann Arbour, MI United States; 6 Cardiology Division, Department of Medicine University of Massachusetts Chan Medical School Worcester, MA United States; 7 Center for Behavioral Science Research Henry M. Goldman School of Dental Medicine, Boston University Boston, MA United States

**Keywords:** mobile application surveys, mixed methods, electronic data collection, mHealth, mobile health, mobile application, mobile applications, app, apps, application, applications, digital health, digital technology, digital intervention, digital interventions, smartphone, smartphones, usability, usable, usableness, usefulness, utility, health information

## Abstract

**Background:**

Studies evaluating the usability of mobile-phone assessments in older adults are limited.

**Objective:**

This study aims to identify design-based barriers and facilitators to mobile app survey completion among 2 samples of older adults; those in the Framingham Heart Study and a more diverse sample from a hospital-based setting.

**Methods:**

We used mixed methods to identify challenging and beneficial features of the mobile app in participants from the electronic Framingham Heart Study (n=15; mean age of 72 years; 6/15, 40% women; 15/15, 100% non-Hispanic and White) and among participants recruited from a hospital-based setting (n=15; mean age of 71 years; 7/15, 47% women; 3/15, 20% Hispanic; and 8/15, 53% non-White). A variety of app-based measures with different response formats were tested, including self-reported surveys, pictorial assessments (to indicate body pain sites), and cognitive testing tasks (eg, Trail Making Test and Stroop). Participants completed each measure using a think-aloud protocol, while being audio- and video-recorded with a qualitative interview conducted at the end of the session. Recordings were coded for participant usability errors by 2 pairs of coders. Participants completed the Mobile App Rating Scale to assess the app (response range 1=inadequate to 5=excellent).

**Results:**

In electronic Framingham Heart Study participants, the average total Mobile App Rating Scale score was 7.6 (SD 1.1), with no significant differences in the hospital-based sample. In general, participants were pleased with the app and found it easy to use. A large minority had at least 1 navigational issue, most committed only once. Most older adults did not have difficulty completing the self-reported multiple-choice measures unless it included lengthy instructions but participants had usability issues with the Stroop and Trail Making Test.

**Conclusions:**

Our methods and results help guide app development and app-based survey construction for older adults, while also giving consideration to sociodemographic differences.

## Introduction

Adults aged 65 years and older are increasingly using tablets and smartphones and engaging with a range of technologies [1]. Technology use can reduce social isolation [2] and enhance communication with family members and health care providers, thereby, increasing well-being. Further, digital technologies have the potential to improve the health of older adults by facilitating symptom monitoring and self-care management as well as monitoring cognitive and mobility decline. However, older adults often lack confidence in their ability to use technology [3] and report needing assistance with new electronic devices and mobile apps [4]. They face unique challenges with technology including poor eyesight, hearing loss, fine motor skill and sensory limitations, and cognitive decline. These challenges make it essential to understand how technologies can be made more useful to older adults. Perceived value, usefulness, and impact on quality of life are important predictors of technology adoption in this age group [5,6]. In addition, a design that minimizes user frustration will enhance the use and lower the risk of leaving older users out of the technology revolution.

Older adults are often not well represented in user testing of technology [7] due to the restricted age range of research studies, physical or sensory impairments, or because technology studies may be less appealing to them. There are a growing number of smartphone apps that include opportunities for self-management of specific diseases and cognitive self-assessment but the quality and usability of the apps are often unknown especially among groups of older adults and adults from diverse race and ethnic populations [8,9]. In addition, health care providers and hospital systems are increasingly requesting that patients complete previsit health questionnaires electronically, which help with care efficiency and are preferred by providers [10,11]. However, older adults are less likely to access and use patient portals and may have unique needs influencing their use [12-14].

Usability information provides practical recommendations that can help increase patient responsiveness to electronically collected data. For example, studies that have evaluated the usability of mobile apps that assess fall risk demonstrated the importance of simple instructions and clear feedback such as a color change to indicate task completion [6,15,16] A mobile app designed for older adults with heart failure to report Patient Reported Outcomes Measurement Information System (PROMIS) measures demonstrated that these adults successfully returned the PROMIS data and an additional survey indicated high levels of usability [17].

We designed a smartphone app for use by community-dwelling older adults who are participants in the Framingham Heart Study (FHS). The smartphone app consists of surveys with different response formats (eg, multiple choice, pictorial, and tasks). The aim of this study is to identify design-based barriers and facilitators to mobile app navigation and survey completion through usability testing. We also sought to understand whether participant feedback differed depending on the response format of each measure. Because FHS participants were White, we enrolled a diverse sample of older adults at a second site to understand if any additional barriers to mobile app survey completion were observed given that digital literacy and preferences for using technologies can vary across older adults of different races and ethnic backgrounds [12,18]. Importantly, little is known about the usability of mobile apps for racially or ethnically diverse populations [8].

## Methods

### Study Design

**Figure 1:**
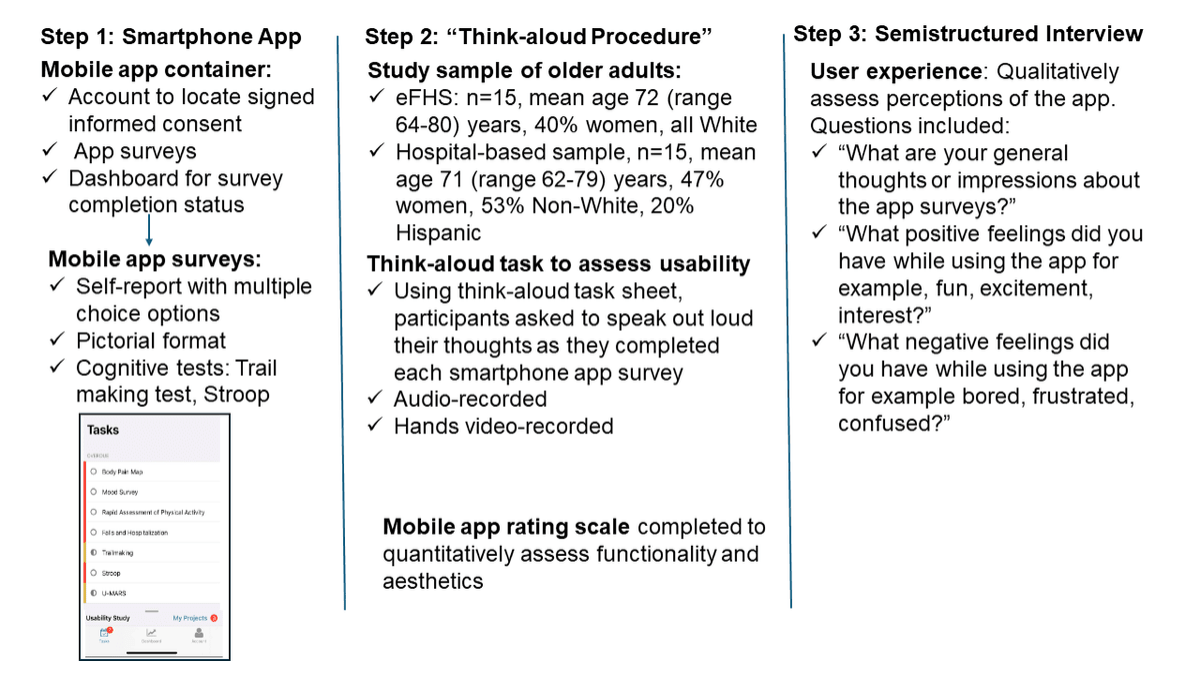
Overall study design.

The study used mixed methods to conduct 1 usability testing session followed by a postsession interview with enrolled participants (Figure 1). Usability testing methods included using the “think-aloud” protocol while conducting a series of surveys and tasks on the smartphone app. This was followed by a semistructured interview using an interview guide, to solicit information on barriers encountered in the session.

### Study Sample

The study sample was drawn from 2 sites: the FHS Offspring study and a hospital-based site. The FHS Offspring participants were recruited in 1971 and are invited back to the research center for examination every 6 to 8 years [19]. The tenth examination of the FHS Offspring participants occurred from 2019 to 2022 and included a mobile health component called the electronic Framingham Heart Study (eFHS). For this study, we enrolled eFHS participants who were English-speaking, owned a smartphone (iPhone or Android), attended Offspring exam 10 before the eFHS began, and enrolled in eFHS between July and September 2022. The eFHS research technician assisted the participants with registration, informed consent, and app download. Because most of the eFHS sample had iPhones and were less racially and ethnically diverse, we recruited participants from a second site who were not part of eFHS. The second site was a hospital in an urban area with a racially and ethnically diverse patient population. At the second site, inclusion criteria were age 60 years and older, English speaking, and able to attend a study session between December 2022 and April 2023. Participants were recruited through flyers placed in clinic waiting rooms and community centers, participation in prior research studies, and through patient registry lists. Non-White and Hispanic/Latino patients were oversampled from patient registry lists. As with eFHS, the research technician assisted the participants with registration, informed consent, and app download on a study iPhone. Participants at both sites were using the app for the first time. We enrolled 15 participants at each study site to ensure the representation of men and women, iPhone and Android users, and older people below and above 75 years. In addition, the number of unique challenges identified with the study design proposed appears to asymptote 15 participants [20]. While eFHS participants were not compensated for their time in the study (because FHS participants have not been compensated for participation in the parent study), participants from the hospital-based cohort were provided a US $100 card for participating upon completion of the session.

### Ethical Considerations

The institutional review board at Boston University Medical Campus approved the eFHS study (H-36586) and this study (H-42659). The institutional review board at the University of Massachusetts Chan Medical School approved the hospital-based study (approval number 00000567).

### Measures

#### Study App

A mobile app hosted a compendium of different types of survey assessments and tasks that users could click on to complete. CareEvolution’s MyDataHelps Designer platform was used to build the smartphone app surveys for iPhone (iOS 10.0 or higher) or Android (version 7.0 or higher) devices. The MyDataHelps mobile app container includes an account where participants can locate their signed consent form, tasks (app surveys), and a dashboard. The dashboard was created in the app to provide the participant with survey completion status and encouragement with a thank you message. Investigators and CareEvolution industry partners internally tested the app surveys and tasks with attention to consistency inspection, and user-centered design principles to ensure clear instructions, easy navigation, and simple words and sentences [21].

Because the goal of the study was to assess usability, we included a variety of app-based surveys and tasks with different response formats (Multimedia Appendix 1). First, we tested several self-report surveys with multiple choice options including (1) the short form of the PROMIS measure of mood (anxiety and depression, 8-items) [22] and cognitive function (4-items) [23] that included 5 response options that ranged from “never” to “always” for the mood assessment and “not at all” to “very much” for the cognitive function assessment; (2) falls and hospitalizations with yes/no response options and a calendar wheel to ascertain the date of a fall occurrence; and (3) rapid assessment of physical activity [24] to report the frequency of physical activity and the level of intensity (light, moderate, and vigorous), assessing frequency, intensity, and time, with yes/no response choices. The hospital-based site did not complete the PROMIS measure of mood. Second, we tested a pictorial format measure to collect data on chronic pain, based on a modified version of the Michigan body pain map measure [25]. Participants were shown an outline of a human body and were asked to click on the different places of the body where they are currently experiencing chronic pain, defined as pain lasting 3 months or longer. The app first displays the image of the front of the body and next the back of the body. If the participant is not experiencing chronic pain, a box was provided so that the user can indicate “no chronic pain.” The third and final smartphone tasks tested were 2 commonly used cognitive tests. The Trail Making Test [26,27] is a timed assessment that requires the user to consecutively tap dots in alternating order between numbers and letters by first tapping the number “1” followed by tapping the letter “A” and then “2” followed by “B” until the user reaches the number “7.” Correct answers result in the appearance of lines between the dots, resulting in a “trail.” Finally, we assessed the Stroop [28] on the smartphone that requires the user to complete a series of 4 increasingly more difficult tasks responding as quickly and accurately as possible to changes in color and instructions. Because the Stroop requires the user to be able to see colors (yellow, green, red, and blue), persons with color blindness are not eligible for this task. A practice session was provided for each set of the 4 tasks with the ability to repeat the practice session should the user desire to do so [29]. At the end of the testing session, participants completed the Mobile App Rating Scale (MARS) on the smartphone to assess app functionality and aesthetics including ease of use, navigation, visual appeal, performance, graphics, and layout [30]. Items were rated by the participant using a 5-point Likert scale from 1=inadequate to 5=excellent.

Demographic data were not collected within the study app. For eFHS participants, these data were collected as part of Offspring exam 10. Sociodemographics such as age, gender, and employment were assessed via the research study coordinators and entered into a secure web-based software platform, REDCap (Research Electronic Data Capture; developed initially at Vanderbilt University, now collaborative support from the REDCap consortium) at the hospital-based site.

#### Procedure

Study sessions were conducted in person at the FHS Research Center. eFHS participants were asked to complete the above assessments using their own smartphones, but hospital-based participants used a study smartphone (iPhone 7). In addition, they were also asked to navigate to different areas of the MyDataHelps app container (account, tasks, and dashboard). While doing these tasks, participants were asked to “think your thoughts” out loud, including feelings (positive like “fun” and negative like “frustrating”). Prior to beginning the usability testing, the research technician demonstrated the “think-aloud” procedure using the text app on a smartphone and then asked the participant to demonstrate the think-aloud procedure using the same app verbalizing every movement, feeling, and decision. Once the participant was ready to begin, the research technician encouraged the participant to use the think-aloud task sheet (Multimedia Appendix 1) that included a list of app-based tasks. Participants were asked to speak out loud their thoughts, feelings, and actions as they completed each task. The participant was audio-recorded, and the participant’s hands were video-recorded throughout the think-aloud procedure. The participant also completed the MARS [30] on the smartphone after the think-aloud procedure. The technician was present to audio- and video-record the think-aloud procedure and to encourage participants to speak their thoughts out loud. The technician was explicitly trained not to assist the participant with the app unless the participant was irrevocably stuck.

After completion of the think-aloud procedure, the research technician conducted a 15-20–minute interview using open-ended questions and reflective listening to obtain participant feedback on their experiences using the app. Interview questions are available in the Multimedia Appendix 1 and include questions such as “What are your general thoughts or impressions about the app surveys?”; “What positive feelings did you have while using the app for example, fun, excitement, interest?”; and “What negative feelings did you have while using the app for example bored, frustrated, confused?” In addition, the interviewer asked the participants to what extent family, friends, and people of their own age would be able to use and enjoy the app and their thoughts on how to ensure the app would be acceptable to people of different cultures. The research technicians (AD, NA, and AH) are coauthors of this work.

#### Research Technician Training

In total, 4 interviewers were trained (by BB and JF), 3 were bachelor level and 1 was PhD level. Training consisted of learning the think-aloud procedure and also how to conduct the post think-aloud interview. Building rapport and communication skills (open-ended questions and reflective listening) were part of the training. Training included didactics and role plays and trainees were required to complete 3 sessions with “mock” participants, supervised by 1 or both trainers before being cleared to do the protocol with study participants. Feedback on study participants was provided to research technicians on an ongoing basis, by viewing the audio and video tapes together. The audiotaped portion of the think-aloud procedure and interview was professionally transcribed (Daily Transcription).

#### Process of Coding the Sessions

Investigators developed a coding sheet and accompanying coding manual for use by teams of coders when coding the video- and audio-recordings of the think-aloud procedure and postinterviews. The coding sheet included general items (eg, navigation between surveys, tapping in the wrong area to advance to the next task, and unclear instructions for surveys) as well as assessment-specific variables (clarity of instruction, understanding concepts, navigating within a survey, and “look and feel” eg, font size, line spacing, and color). For training purposes, all coders reviewed 3 participant recordings together and resolved any coding discrepancies before separating into the 2 coding teams to code in pairs. In order to maintain coding reliability over time, the 2 teams (team one: JF, JM; team 2: BB, DDM) independently coded the same participant recordings on 5 occasions throughout the coding process and came together to review the coding sheets for any discrepancies across teams to ensure all coders were following the coding guidelines. The average percent agreement between coders ranged from 80.5% to 98% across the 5 recordings that were coded in common by the 2 teams.

#### Statistical Analysis

Descriptive statistics of the 2 study samples used mean and SD for continuous variables, and numbers and percentages for nominal variables. For the comparison of continuous variables, 2-tailed *t* tests were applied, and chi-square tests or Fisher exact tests were used to compare nominal variables between the samples. The percent agreement between coding teams was calculated by the ratio of the number of discrepancies divided by the number of items in the coding sheet. For the MARS, all items used a 5-point Likert scale and the mean score for each domain (functionality and aesthetics) was calculated separately and an overall MARS score was computed for each of the 2 study samples. In addition, we calculated the mean score of each item within a domain (eg, layout, graphics, and visual appeal).

## Results

### Study Sample

In eFHS, 15 participants signed informed consent (mean age of 72, SD 4.2, range 64-80 years; 6/15, 40% were women; 1/15, 100% non-Hispanic, White). All eFHS participants owned a smartphone with 9 of 15 (60%) of the eFHS study sample participants owning an iPhone (Table 1). In the hospital-based site, 77 were contacted, 19 declined to participate, 3 deferred enrollment to a later date, and 15 participants signed informed consent (mean age 70.6, SD 6.2, range 62-79 years; 7/15, 47% women; 3/15, 20% Hispanic/Latino; and 8/15, 53% non-White). In the hospital-based sample, 1 participant did not own a smartphone, and among smartphone owners, 6 of 14 (42.9%) owned an iPhone. More than half of the participants at both sites had a college education or advanced degree. While nearly 90% (13 of 15 participants) of eFHS participants reported their health to be very good to excellent, only one-third (5 of 15 participants) of the participants at the hospital-based site did (*P*=.003).

**Table 1:** Participant characteristics.

Characteristics		eFHS^a^ (n=15)	Hospital-based sample (n=15)	*P* value	
**Age (years)**					.48
	Mean (SD)	72 (4.2)	70.6 (6.2)		
	Range	64-80	62-79		
Women, n (%)		6 (40)	7 (46.7)	.71	
Non-White, n (%)		0 (0)	8 (53.3)	.002	
Hispanic, n (%)		0 (0)	3 (20)	.22	
**Smartphone owner, n (%)^b^**					
	iPhone	9 (60)	6 (42.9)	.36	
	Android	6 (40)	8 (53.3)	.36	
Bachelor degree and higher, n (%)		10 (66.7)	9 (60)	.71	
Marital status (married, living as married), n (%)		12 (80)	8 (53.3)	.12	
Income <US $55,000, n (%)^c^		4 (31)	5 (33)	.89	
Subjective health, very good or excellent, n (%)		13 (86.7)	5 (33.3)	.003	

^a^eFHS: electronic Framingham Heart Study.

^b^All offspring participants owned a smartphone (iPhone or Android); 1 hospital-based participant did not own a smartphone.

^c^Income of 2 participants in eFHS sample was unknown.

In the eFHS sample, the average length of time of the think-aloud procedure was 25.5 (range 12.4-44.0) minutes and the postprocedure interview time on average was 18.7 (range 12.4-20.9) minutes. Two participants declined the interview (think-aloud times were 28.5 and 33.35 minutes, respectively). At the hospital-based site, the average length of time of the think-aloud procedure was approximately 11 minutes longer (mean 36.5, range 24.1-55.1 minutes) while the postprocedure interview time was similar to eFHS with an average of 18.5 (range 10.6-35.1) minutes. No participant at the hospital-based site declined the post think-aloud interview.

### Barriers to App Use Identified During Think-Aloud and Postprocedure Interview

Table 2 presents the themes identified from the think-aloud task and postprocedure interview along with sample participant quotes.

**Table 2:** Barriers and facilitators identified during the think-aloud and postprocedure interview.

Domain or survey type	Theme	Sample quotes
General structure, satisfaction	Information about security of app Use to raise awareness of health Everyone could learn from it	“The more I do, the easier it gets” “I just think it’s gonna help me monitor my health and go from there. I like technology that helps me and doesn’t just amuse me or keep going and if this helps me stay healthy, stay fit, it makes sense.”
Ease of use	Easy, simple, fun Questions easy to answer Confusion related to not paying attention and not reading the app instructions carefully enough Font color yellow difficult to see Font could be bigger	“The app spells everything perfectly clear after you read it a couple times to get it” “I wasn’t really paying attention to what I was reading. That was mostly my problem.”
Navigation	Navigating within and between surveys was easy More detail on getting back to the home screen and using “next,” “cancel,” and “done”	“The more I did it, the more I was able to figure out how to get from one place to another.” “You might want to give a little more detail about some of the sections, like what happens when you cancel, what happens when you hit done.”
Multiple-choice survey formats	Surveys were interesting and simple except physical activity survey with lengthy definitions and confusing response choices	“It was easy the questions were simple and it was easy to find the answers.”
Pictorial format	Body pain map did not include all areas that can be a real problem Checkbox worked differently than response choices for other surveys Hard to find the “no pain” box—small font	“Well, on the pain thing, I think they should have something near the anus. Because that can be a real headache. And then, of course, for women, it would also include, uh, the uterus.”
Cognitive tasks: Trail Making Test and Stroop	Stroop was confusing Difficulty understanding practice session versus testing session Confusion over 4 increasingly challenging sets of tests within the Stroop task TMT^a^ had difficulties with instructions and navigation but the eFHS sample did not	“The all underling thing was a little confusing to me. I think because I was trying to get through it quick. I lost track of whether underline the word or is it the color or is it this color or the word?” “The trail-making one I liked”
Friends or family	Fear of technology; Little interest in learning how to use technology Learning curve for older people	“I think they would find it very useful I think it’s very useful, just for like, the cognitive part of it.”
Different cultural backgrounds	Need the app to be available in different languages	“I come from the Indian community, and we place a lot of value on education, you know, I think this is the kind of thing that, you know—I would—we do—I would like to be quizzed, and this is a quiz.” “For example, I came from Burma. Burma used to be very underdeveloped country, and, also, still lots of problem. But they are very good at technology.”

^a^TMT: Trail Making Test.

#### General Structure of the App

In general, participants were pleased with the general structure of the app. However, a few participants had not used an app previously and they said it was “not intuitive” but “once you run through it a few times it isn’t a problem.” Participants generally thought that the app had the right amount of surveys (and that additional surveys would make them bored). Participants suggested that a progress indicator be inserted to let users know the status of survey completion. One participant also raised the importance of conveying information regarding the security of the app.

#### Ease of Use

Overall, participants found the app “easy to use,” “simple,” and “fun.” One participant said that it looked and functioned similarly to apps he was already familiar with, such as a social security app. A small minority of participants, however, wanted increased font size and more user-friendly colors. One participant was frustrated with the functionality of the app, but he blamed it on using an old phone. There were some suggestions for modifications, such as tailoring the app by age group and disability, so it is not so “one size fits all.” Another participant suggested removing the free text space (which was needed for 1 question) or adding a digital keyboard so it is more intuitive that the person is supposed to type. For example, upon completion of the Stroop task, there was a query (“did you encounter any issues during the task?”) that permitted the participant to freely type in a response.

#### Navigation

A large minority of participants reported at least 1 navigational issue, including navigating within and between surveys and also clicking the incorrect area in the app to navigate to the next screen (eg, navigating to “done”). The vast majority of these errors were committed only once. Participants suggested making the buttons look more like buttons or having them flash when you are supposed to press the button, “...making it extremely obvious what you are supposed to hit.” Participants also suggested that more details should be added, such as what will happen after you hit a button (eg, after “cancel” or “done” is pressed), and more information about what the “icons at the bottom of the app” mean (eg, dashboard). A few participants mentioned that the app should avoid having to scroll to see more information, and instead put all of the information on one page if possible or continue to the next page.

### Response Choice Formats

#### Multiple-Choice Formats

Participants reported that most of the multiple-choice surveys were easy and interesting to complete, with only a few sporadic usability errors by a few participants. For the most part, participants reported that the instructions were easy to understand, with one exception, which was “The Rapid Assessment of Physical Activity” survey, which included lengthy definitions of physical activity levels (eg, mild, moderate, and vigorous activity) which were needed to answer the subsequent questions. Participants were confused by the content and format of the definitions. With regard to the latter, both physical activity intensity level and physical activity frequency in a single question (eg, “I do some light or moderate physical activities but not every week,” yes or no).

#### Pictorial Format

For the pictorial format assessment, (modified version of the Michigan Body Pain map), a small minority of participants noted the checkbox for response worked differently than the other surveys; instead of seeing a checkmark in the response box, the response box if selected was highlighted in color and was confusing for some. Some participants also noted that the map did not include all body areas where pain was present. For some participants, the “no chronic pain” response box was difficult to find due to the small font, and the small font used to label the body parts was also difficult for several participants (but necessary to eliminate scrolling to see the entire body outline). There were no participants who reported difficulty understanding the instructions or concepts. Only a couple of participants did not realize that they had to press “next” to go to the next page to view the back of the body to indicate pain areas there. One participant said that bowels and reproductive areas give people his age a lot of issues and that these areas should be added to the body pain map.

#### Cognitive Testing Formats

In terms of the formats used for cognitive testing, most older participants had some difficulty with the process of completing these measures. In terms of the Stroop, some participants had difficulty understanding that they were in the practice session versus the test session. Participants also thought that they needed to do the testing quickly and were confused if they did not read the instructions for each of the 4 test sets, as each had different instructions. Most participants did not take advantage of the opportunity to repeat the practice session when asked by the app, even when they were confused by the task. The yellow font color for the Stroop was also problematic for a few participants. One participant suggested that voice and animation be used to explain the instructions for the Stroop. For the Trail Making Test, the eFHS sample did not report any difficulties with instructions or navigating within the task, and seemed to have a good understanding of the concepts included in the task. However, a large minority of the hospital-based sample had difficulties in all 3 of these areas. There were no difficulties reported on the look and feel of the task (eg, font, line spacing, and color).

#### Relevance for Friends, Family, and Different Cultures

Participants noted that they knew older people who engaged with technology and others who were not interested in the “electronic age,” did not use a computer or smartphone, and had no interest in learning. One participant said that she had a friend aged 90 years or older who would easily be able to interact with the app and another friend of the same age who would have more difficulty. Participants noted older adults may have a fear of technology, be less confident using technology, and need assistance or a training session given that, for older people who have not used computers or technology, using the app would “be like a foreign language to them.” Participants provided their thoughts on using the app in older adults of different cultural backgrounds. Many noted that the current version of the app is available only in the English language and would need translation to other languages. One participant was from a country that they felt embraced technology, and another participant reported her culture valued education and felt like the app included an educational-like component like a “quiz” which would be viewed positively by her culture.

#### Satisfaction With the App

**Figure 2:**
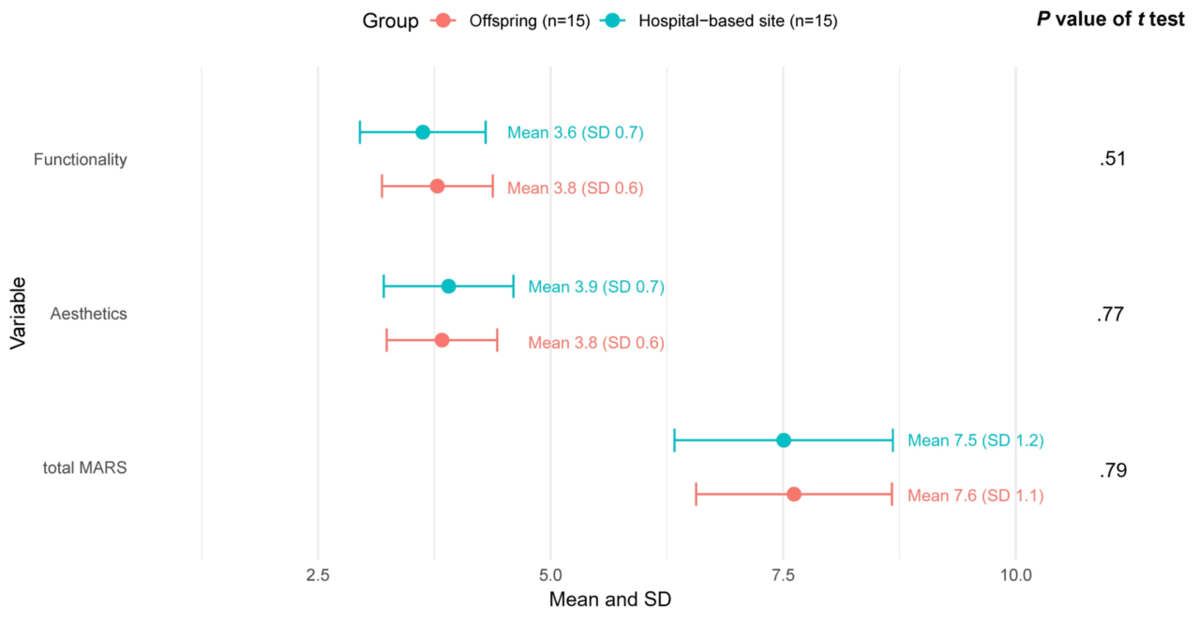
MARS scores by study sample: overall MARS functionality and aesthetics scores. MARS: Mobile App Rating Scale.

**Figure 3:**
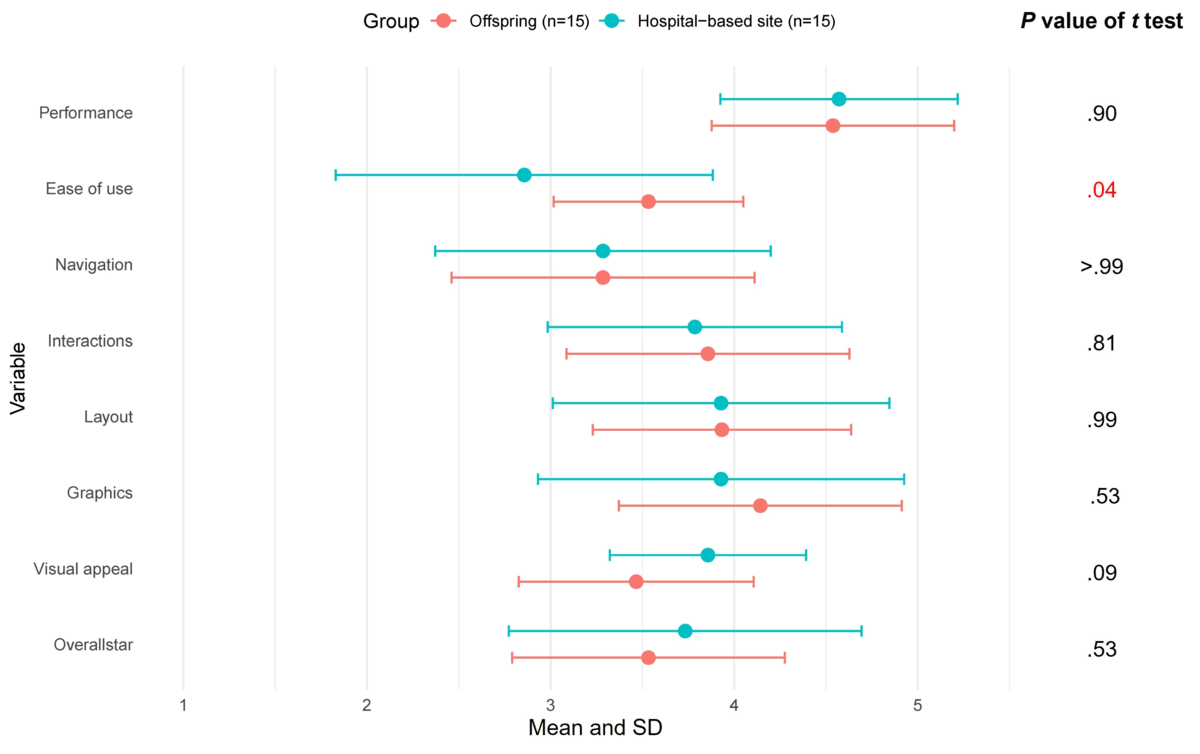
MARS (Mobile App Rating Scale) scores by study sample: individual items within the Functionality and Aesthetics domains.

The mean total MARS score (7.6, SD 1.1), mean functionality score (3.8, SD 0.6), and mean aesthetics score (3.8, SD 0.6) in the eFHS sample did not significantly differ from the hospital-based sample (Figure 2). With the exception of ease of use, the individual items of the functionality and aesthetics scores (performance, navigation, interactions such as taps, swipes, layout, graphics, and visual appeal) did not significantly differ between the 2 samples (Figure 3). Ease of use may have differed as participants at the hospital-based site used a study iPhone whereas eFHS participants used their own smartphone. The performance item was rated the highest with a mean of 4.5 in both the samples. The mean overall star rating was 3.5 (SD 0.7) in the eFHS sample and 3.7 (SD 0.96) in the hospital-based sample indicating participants rated the app above average. In addition, during the interview, participants noted that the app could be used to raise awareness of health and people could learn from it, even though that was not the original purpose of the app. Some participants liked the app because it enabled one to “express yourself” through the surveys. Finally, a few participants suggested making the app “more entertaining” by adding narration.

## Discussion

We tested the usability of a smartphone app designed to collect health information using complementary approaches in a community sample of older adult participants of the Framingham Offspring Study, and to understand generalizability we also tested older adults from a more diverse hospital-based sample. In general, participants liked the structure of the app and found the app was simple, fun, and easy to use. However, a large minority reported navigation issues that mostly occurred once with the ability to learn and figure out how to move within and between app-based surveys. A small minority of participants verbalized a preference for larger font sizes or more user-friendly colors. We observed that most older adults did not have difficulty with the multiple-choice app-based surveys unless the survey included lengthy instructions. Finally, most older adults experienced challenges with the app-based cognitive tasks especially the Stroop which required participants to read and understand a series of 4 increasingly more challenging tests with the Stroop task. Of importance, some participants noted that the app could be used to raise awareness of health and one could learn from it. Our observations confirm those of others that the involvement of older users can result in positive feelings among older adults, dispel stereotypes associated with older users, and the insights gained from older users can be used to enhance the quality of the design [31]. For example, our participants suggested using voice instructions and animated tutorials. To enhance usability, app designers and investigators should consider training that includes tutorials within the app provided by an older adult guide to boost confidence when designing smartphone apps for use by older adults.

This work has several implications for tailoring technology for older adult users. First, a guide within the app explaining the purpose of the app and highlighting key app functions including such functions as “next,” “back,” and “done” would enhance usability. Older adults are more likely to engage with technology that they perceive as useful [5]; therefore, having a clear understanding of the goals of technology is critical. Further, the addition of basic training in smartphone use in older adults less proficient with technology was associated with fewer errors and less cueing during a smartphone app–based health prevention program and may result in improved engagement [32]. In our sample, older adults who had not used an app before did not find it intuitive but after running through it a few times did not find it a problem. Our results support recently published app design guidelines for older adults advocating for initial training, if possible face-to-face, along with video instructions that are contextualized and provide step-by-step instructions to support older users [33]. Training may boost confidence and make the experience as frictionless as possible lowering the potential for abandonment. Second, streamlining and simplifying instructions may enhance understanding by inviting older adults to read them attentively. Participants in our study noted confusion that they attributed to not paying attention or related to the need to slow down and read directions more than once. Consistent with our observation, others have noted the need for clear and simple instructions when designing mHealth apps for older adults [6]. Some participants also requested features they enjoyed in their use of other apps. Gamification of functions, where possible, such as a flashing “done” button or a countdown to the start of the next task may improve engagement. Finally, consider voice narration and animated guides throughout the app surveys where features other than straightforward multiple-choice questions and responses will be encountered.

Our study may have important insights to help address the continued digital disparities observed in older adults. Older adults are increasingly using the internet and smartphones [34]; however, connection to the internet decreases across ages with nearly half of young adults almost constantly connected versus 8% of adults aged 65 years and older [4]. Other key digital health behaviors are also lower in a nationally representative sample of older adults including using health apps, using a digital device to track health or a health-related goal, and digitally communicating with a health care provider [35]. Digital technologies were lifesaving during the COVID-19 pandemic as the rapid transformation from in-person visits to televisits permitted access to health care in a setting that provided social distance and did not expose vulnerable older adults to the virus. Similarly, the ability to participate in digital interventions may provide several benefits, such as improved memory and independent living [36], physical functioning, physical activity [37], depression, and anxiety [38]. Including older adults in technology design and conducting usability testing may address digital health inequities by addressing digital health literacy and creating programs that are user-friendly to this population [39]. They may also improve implementation beyond pilot studies and achieve the needed sustainability of technology solutions [40] for chronic disease management and home care options for older adults and, at the same time, maybe one step in addressing digital disparities. We plan to use the smartphone app more widely in the Framingham cohorts as a tool to monitor health. We will be able to provide critical information on the characteristics of those who enroll and use the technology, as well as those who choose not to.

Our study had several strengths. There is no “best” method to assess usability [21]; therefore, we used both qualitative and quantitative methods. We tested older users with mean age of 70 years and older in both samples often not included in studies testing technology and included older adults from diverse race and ethnic backgrounds. The study sample included a range of older users. Participants without a smartphone or experience with app use and both iPhone and Android users were included. This strategy allowed us to uncover errors with the app beyond what would be observed with “regular” users and permit greater guidance in app redesign to benefit older users.

In addition, participants with health issues were included. Some older adults with health conditions or geriatric syndromes such as frailty have higher levels of nonuse of information communication technologies and more negative views on usefulness and usability [41]. Our study also had some limitations that merit comment. Participants at the hospital-based site used an iPhone only. This may have been a limitation if the participants were Android owners or had a different iPhone version; however, this is also a strength as we were able to include participants who were not smartphone owners. Our observations focus on the first interactions with the app-based surveys. It is beyond the scope of the study to examine other aspects of use such as efficiency (how quickly the survey or task is completed once the design is learned) and memorability (how easy to use after a period of not using the app) that may have important implications to research study designs. Continued engagement with technology changes over time in older adults but the factors related to continued technology use are unclear and require further investigation [42]. Our study took place in Massachusetts, and therefore, may not be generalizable to other geographic areas. Participants with color blindness were not eligible for the Stroop task and were excluded from testing. Therefore, results may not be generalizable to this group of older adults. FHS participants who enroll in eFHS are healthier and have higher levels of education than participants who chose not to enroll.

Our study of a diverse sample of older adults testing several different smartphone app survey types and response formats provides a guide to investigators and clinicians that can be used for future app development and app-based survey construction for older adults. Many older users are able to interact with and enjoy technology. Further work to enhance engagement among older users and diminish digital disparities in this group is needed if the potential of technology to improve well-being, functioning, and health in older adults is to be realized.

## Appendix

Multimedia Appendix 1 Screenshots of app-based surveys and tasks, think- aloud task sheet, and post procedure interview questions.

## Abbreviations

eFHS: electronic Framingham Heart Study
FHS: Framingham Heart Study
MARS: Mobile App Rating Scale
PROMIS: Patient Reported Outcomes Measurement Information System
REDCap: Research Electronic Data Capture
