# Supplementary material for: Smartphone App Designed to Collect Health Information in Older Adults: Usability Study
Source: JMIR Hum Factors. 2024 May 30;11:e56653. doi: 10.2196/56653 (PMC11176875; doi:10.2196/56653)
Supplement: Multimedia Appendix 1 [file humanfactors_v11i1e56653_app1.docx]

**Multimedia Appendix 1. Screenshots of app-based surveys and tasks, think- aloud task sheet, and post procedure interview questions.**

1. Screenshots of app-based surveys and tasks in the Think Aloud procedure 2-32
2. eFHS Think Aloud Task Sheet 3-34
3. Hospital-based Think Aloud Task Sheet 35-37
4. Post-Procedure Interview questions 38-39

**eFHS Survey Screenshots:**

**Mood Survey:**

**
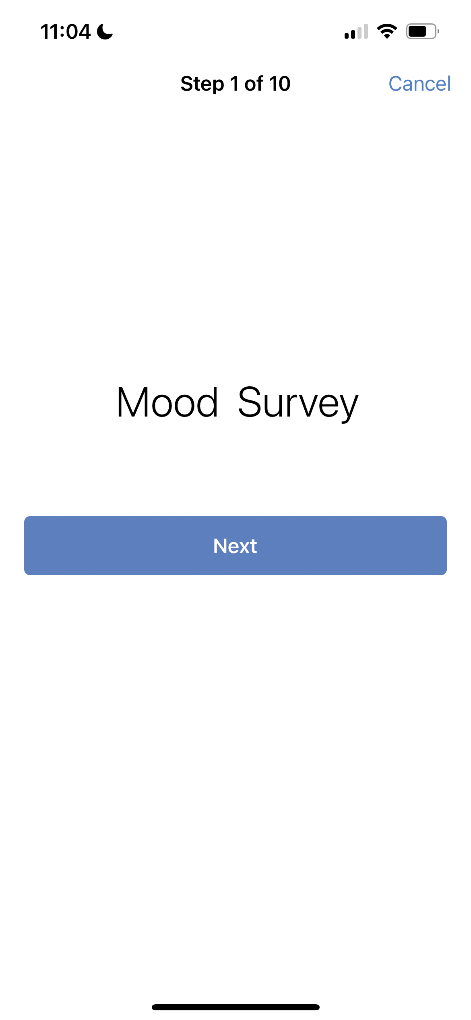

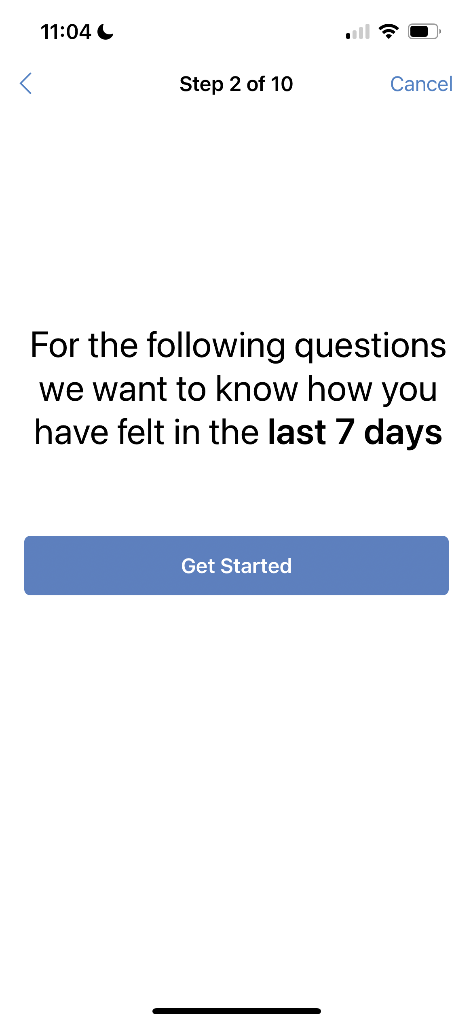

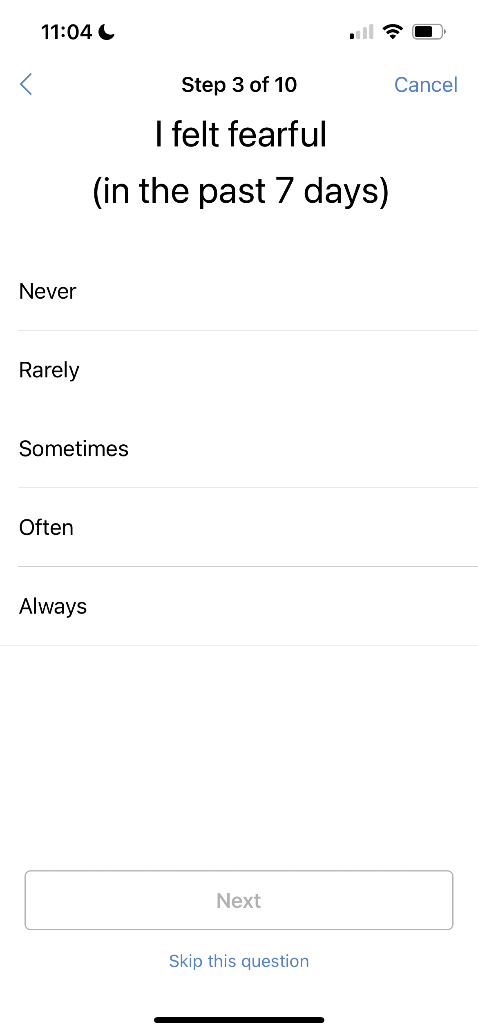

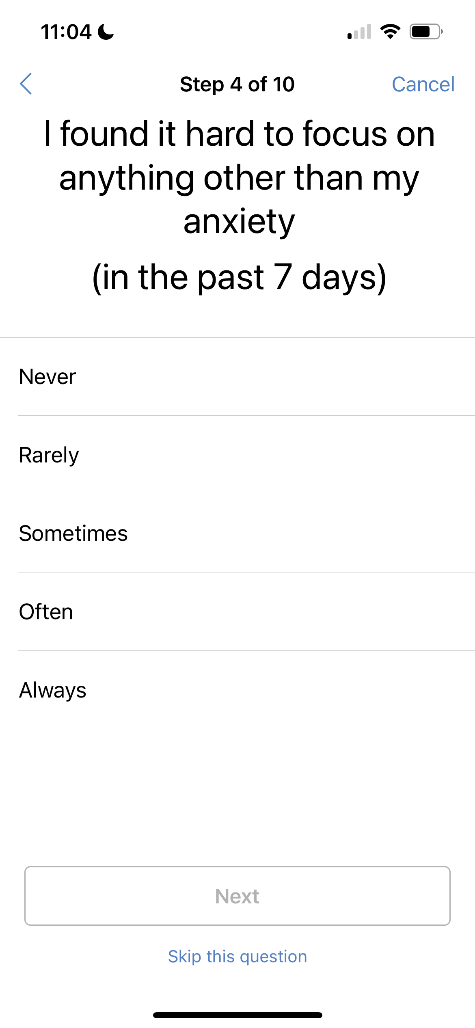

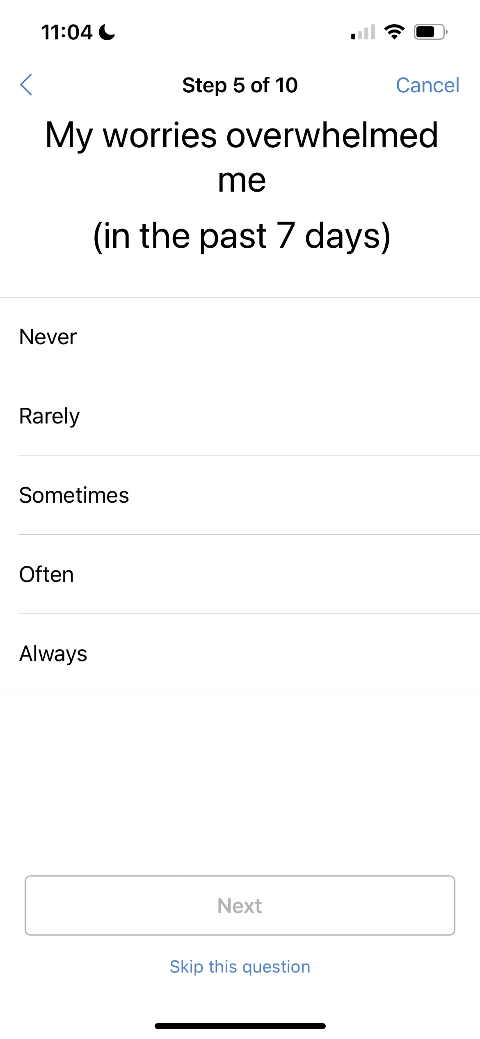

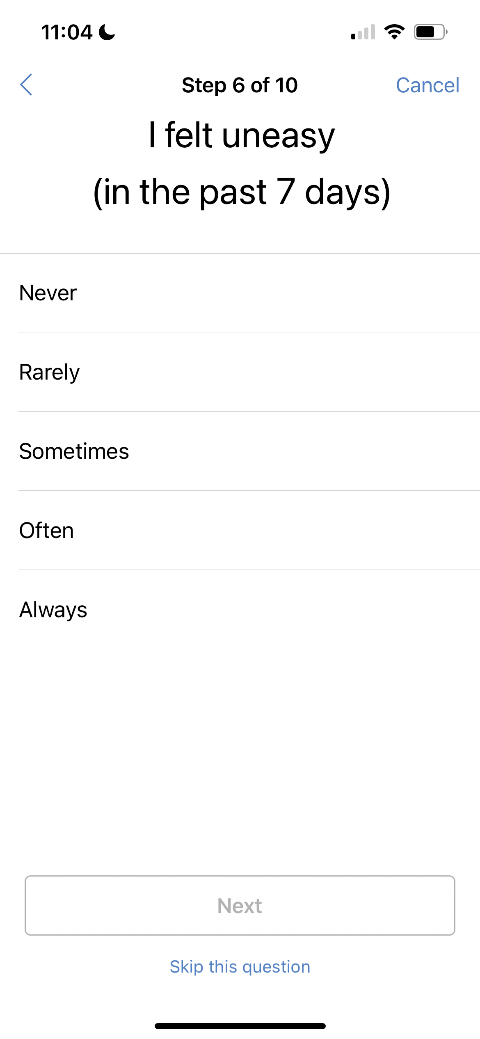
**

**
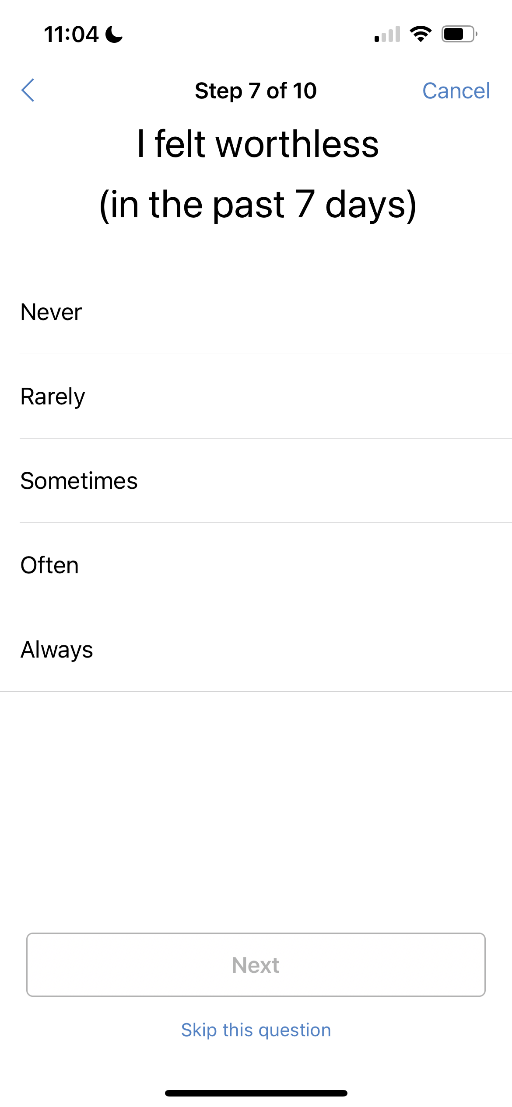

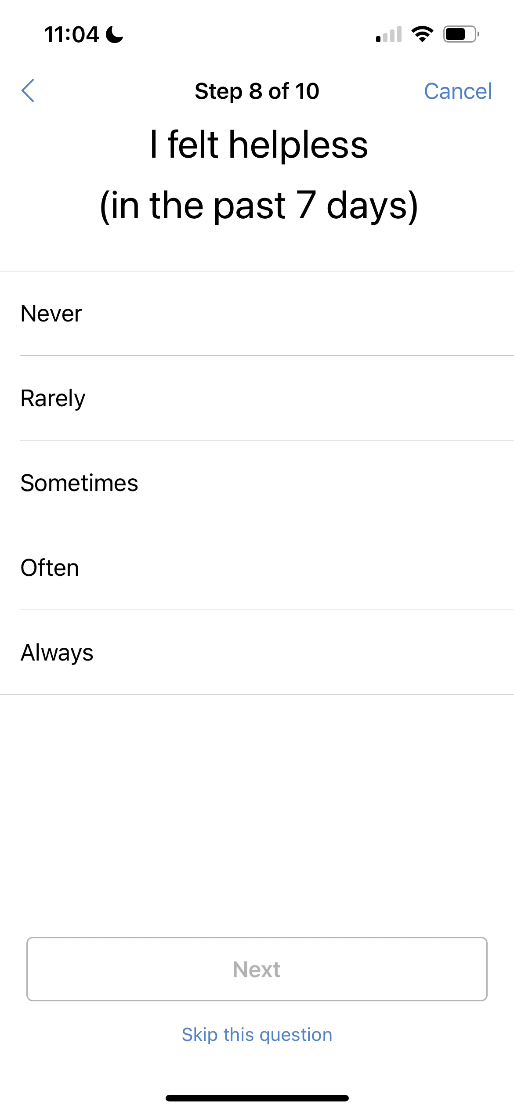
**

**
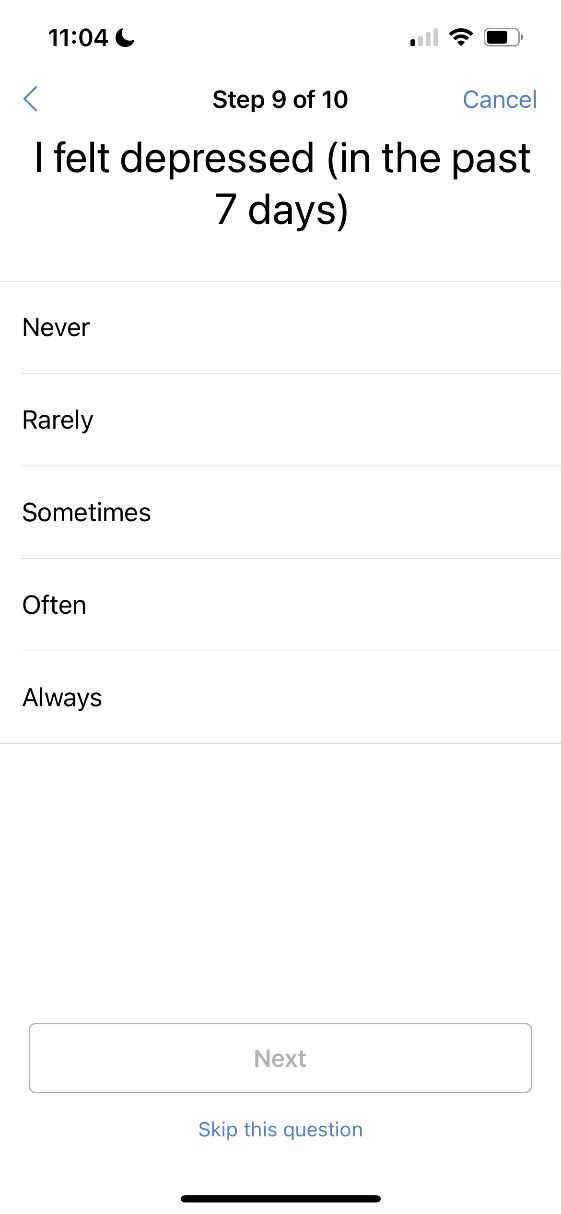

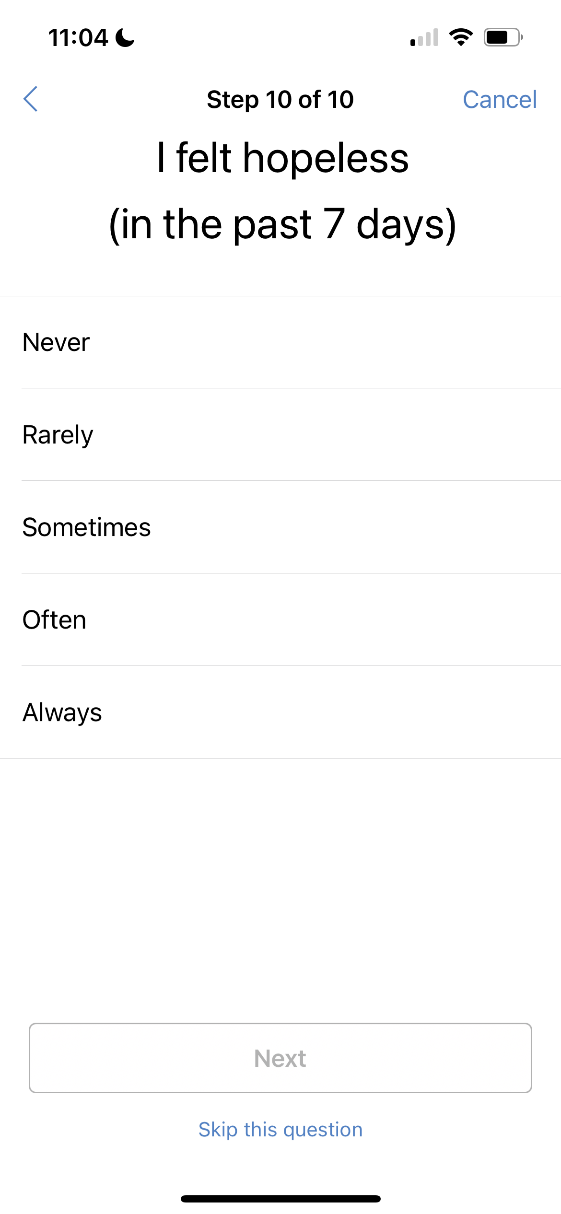
**

**
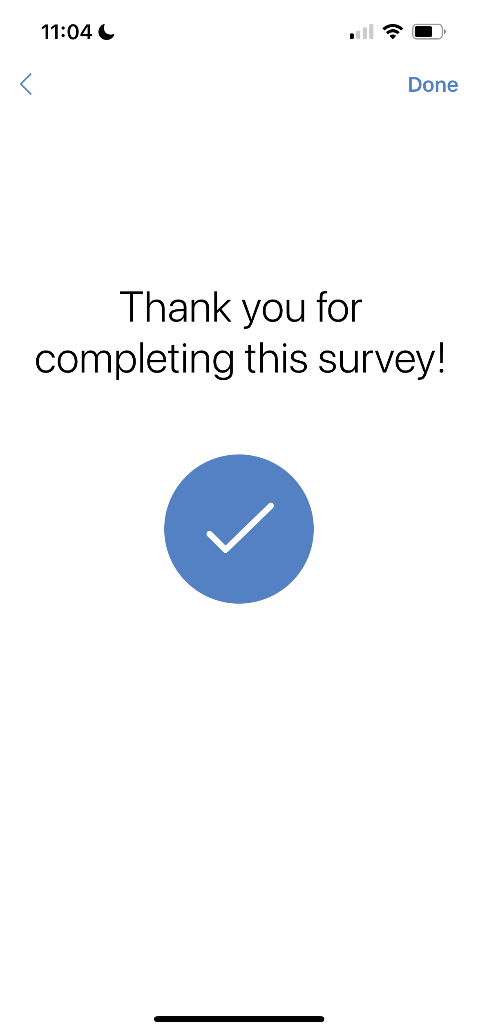
**

**Falls and Hospitalization Survey:**

**
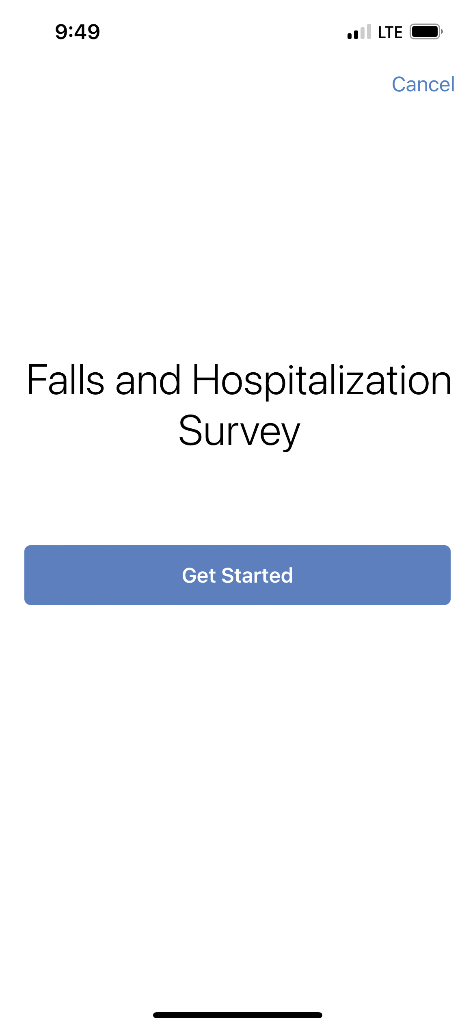
**

**Two options: If No…**

**
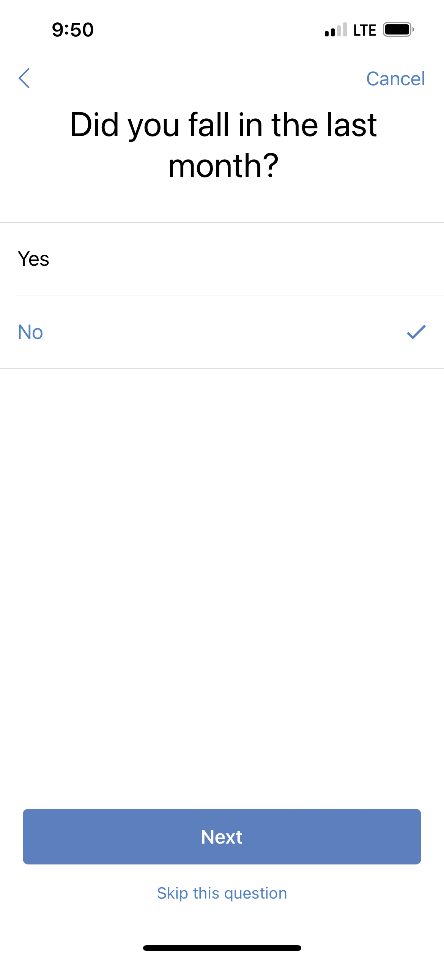

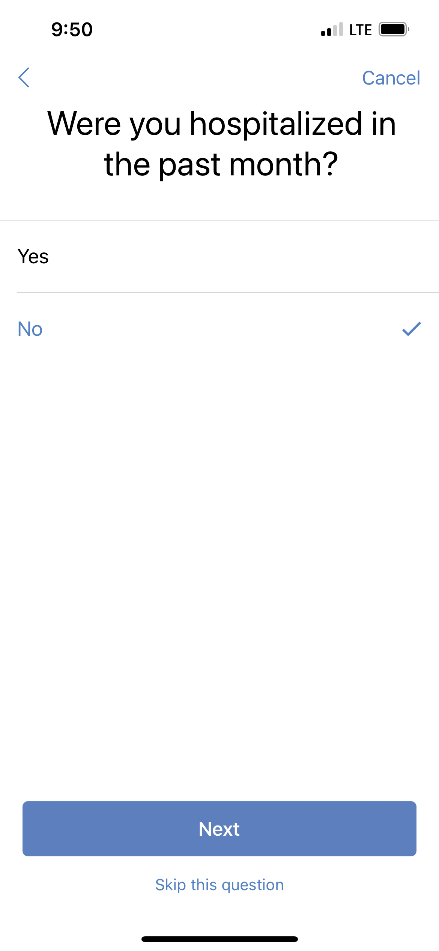

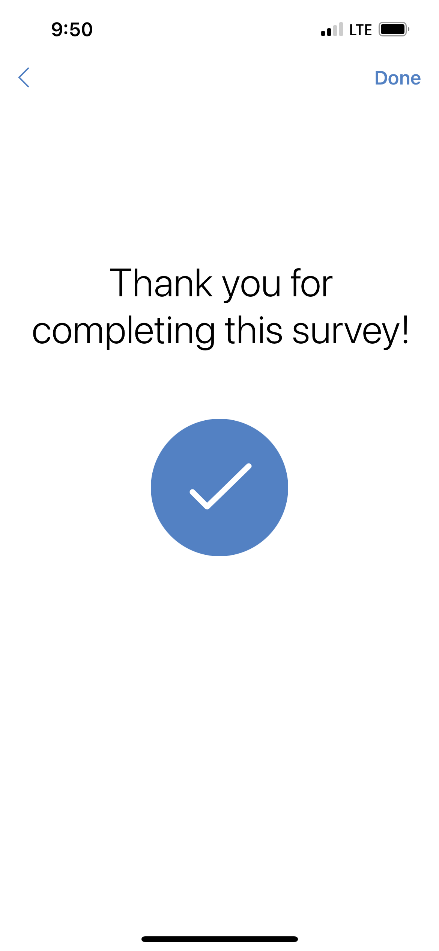
**

**If Yes…**

**
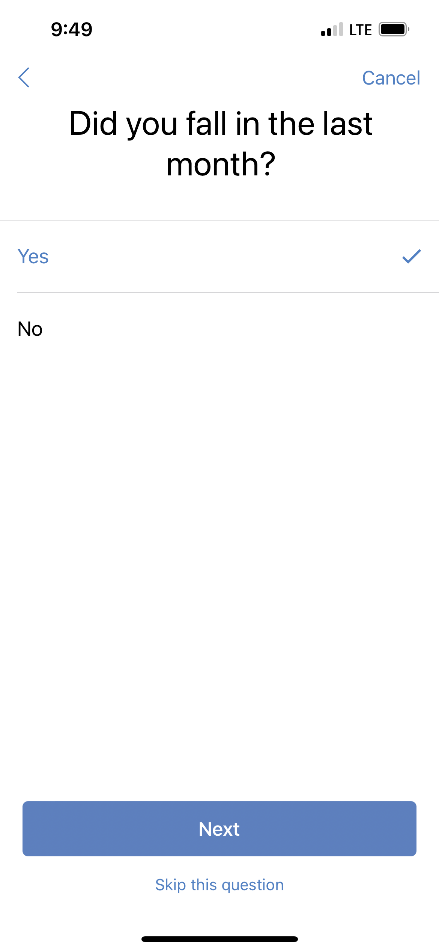

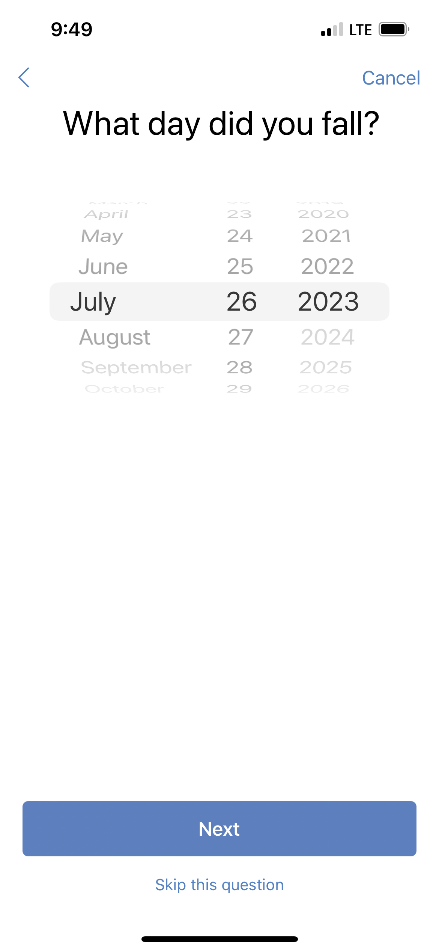

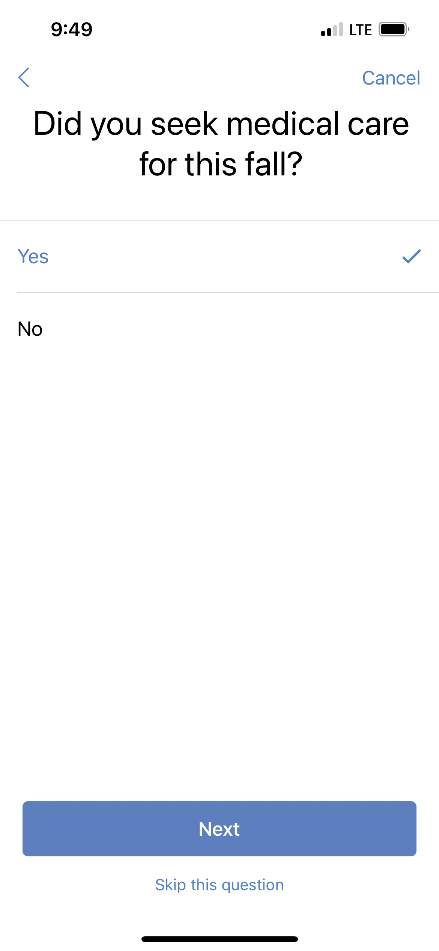
**

**
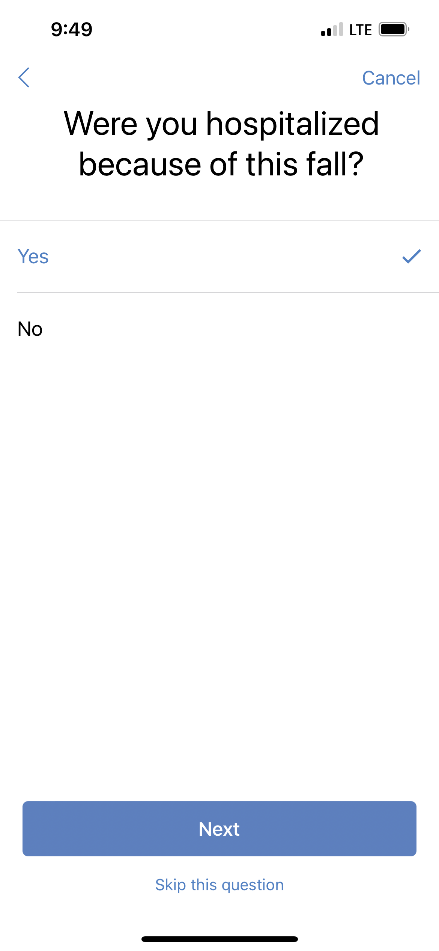

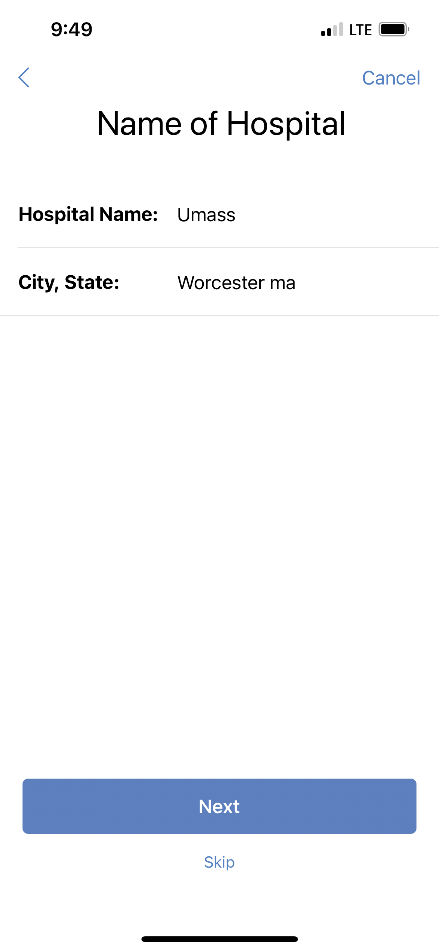

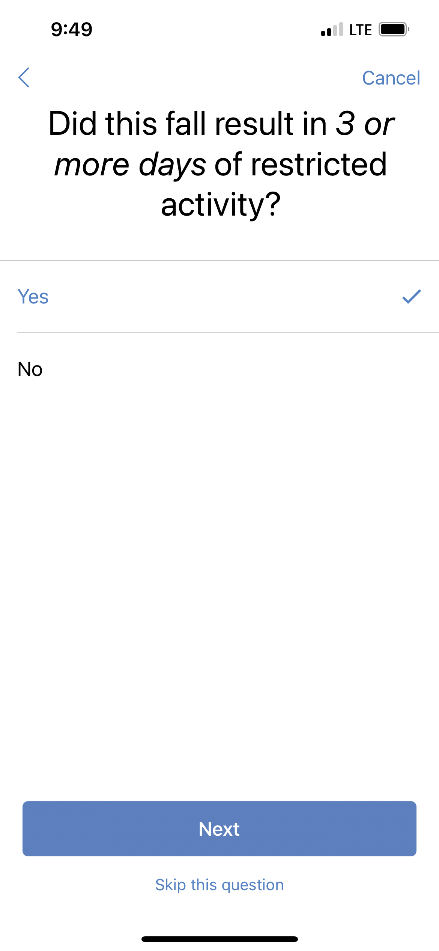
**

**
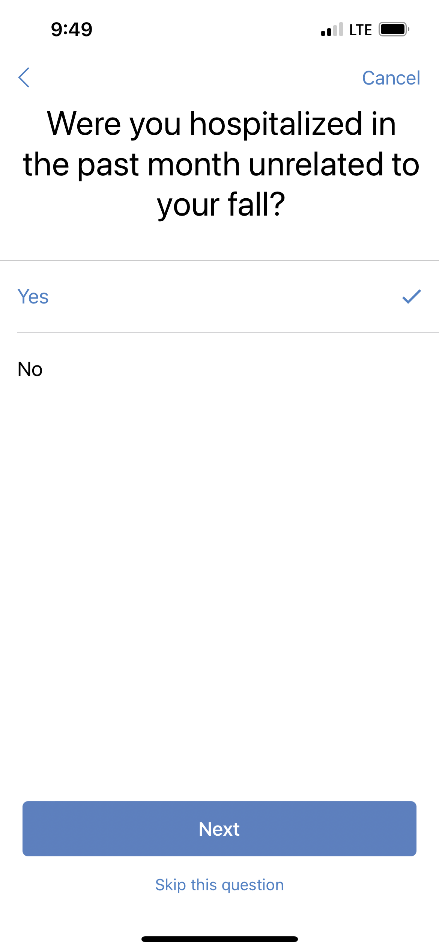

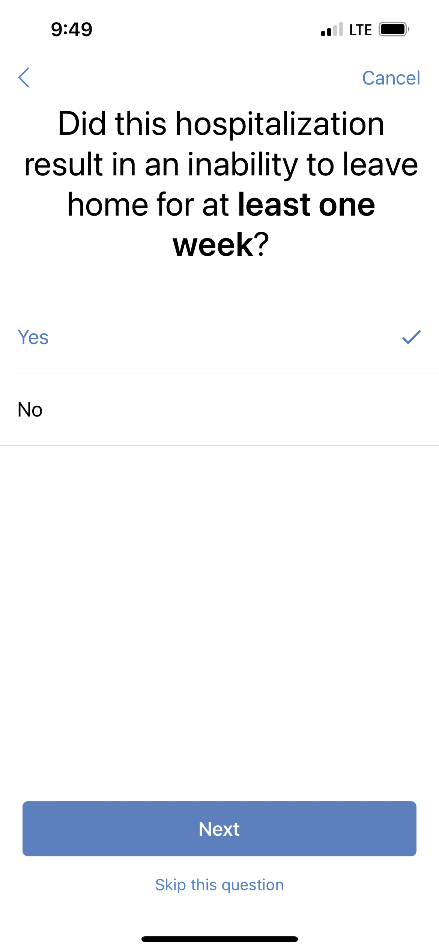

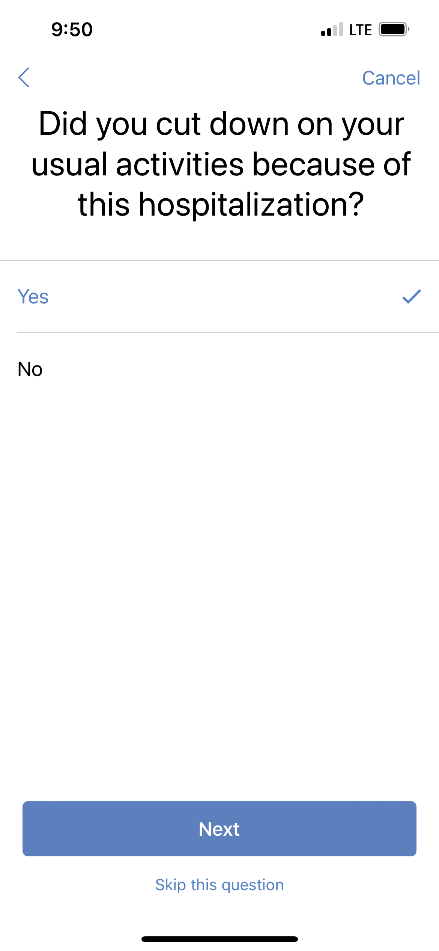
**

**
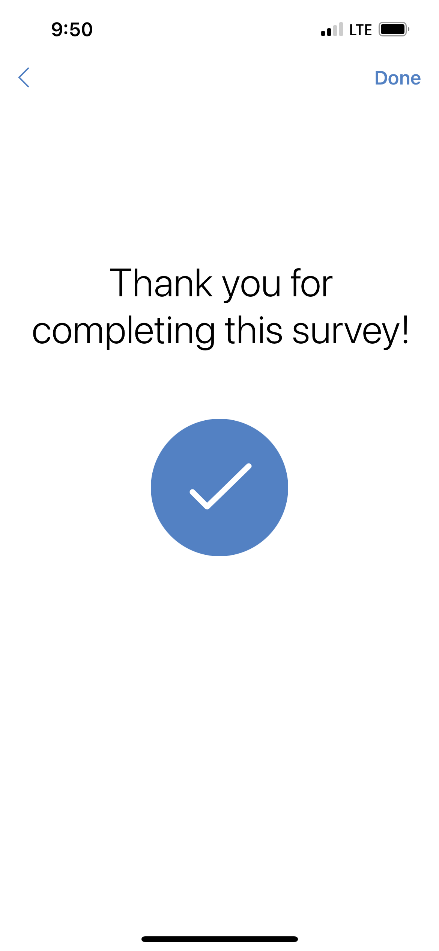
**

**Rapid Assessment of Physical Activity:**

**
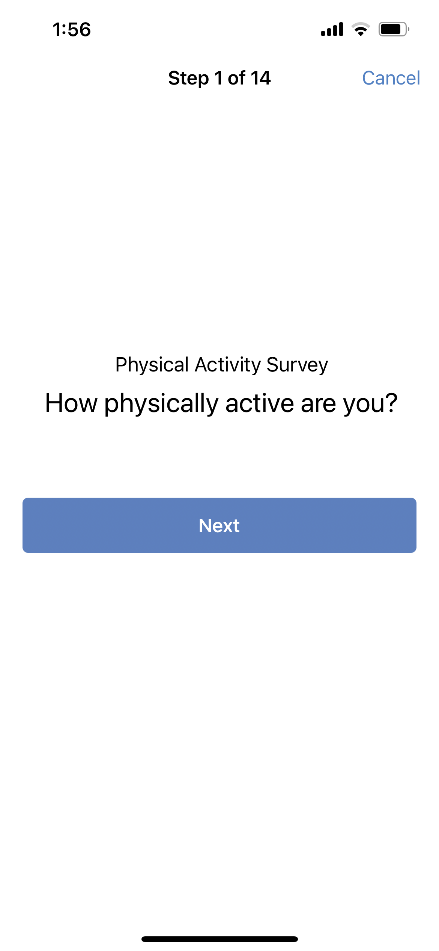

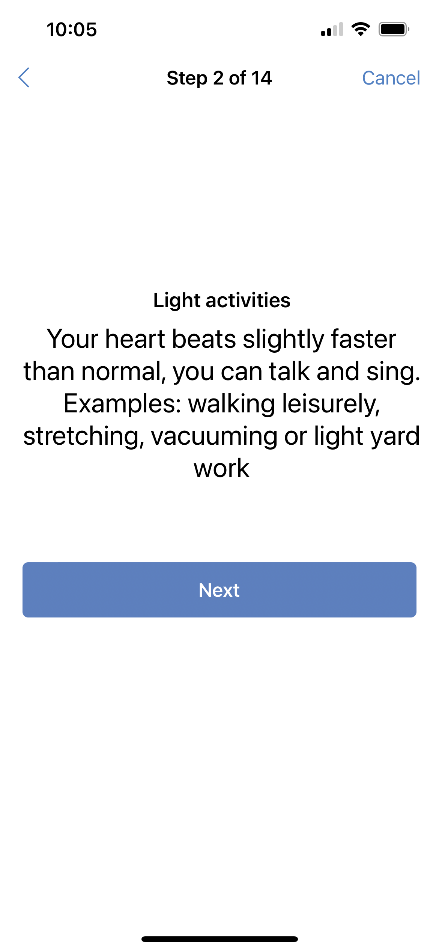

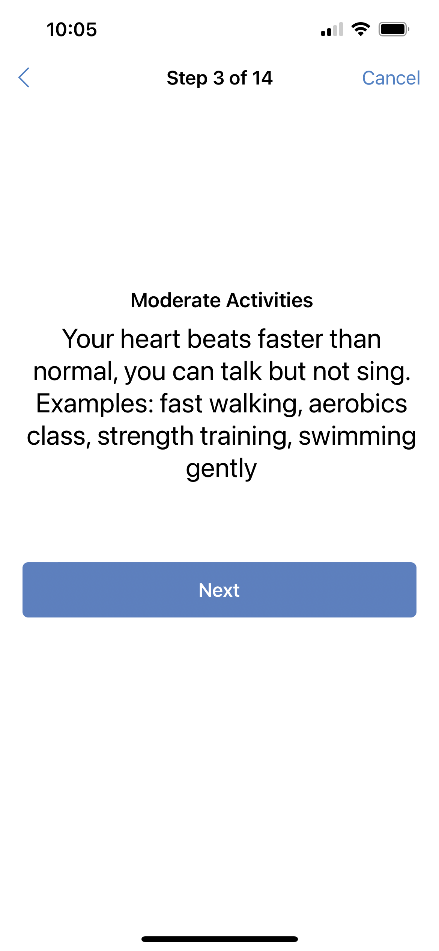
**

**
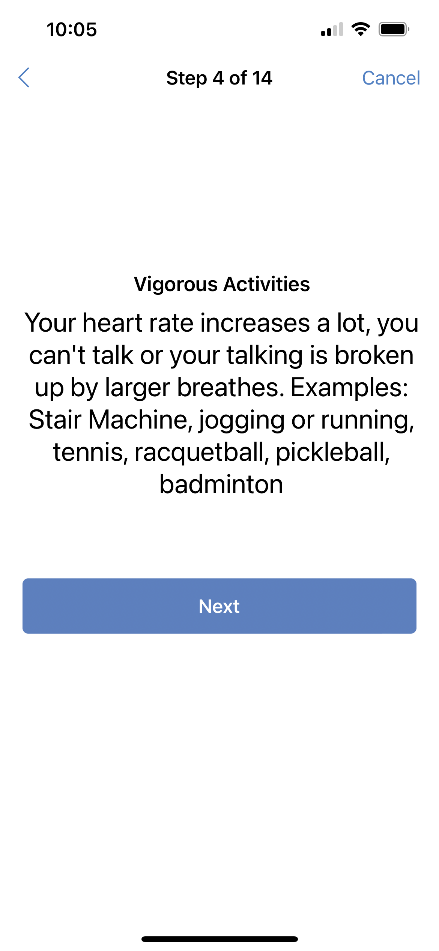

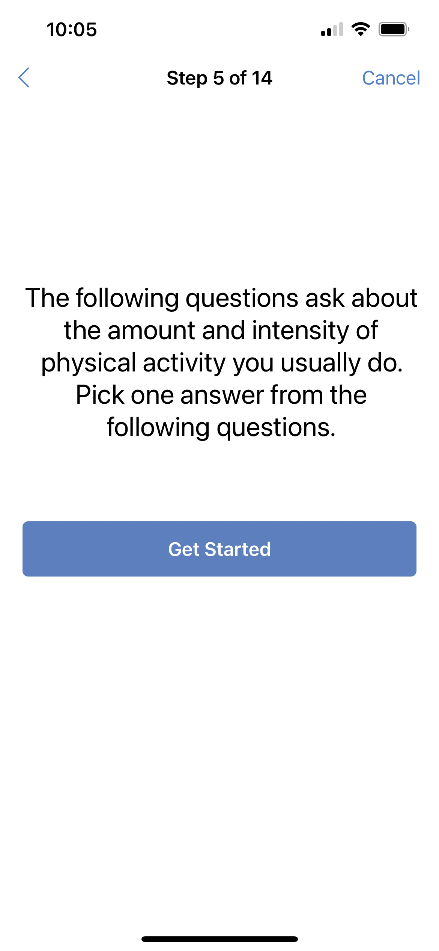

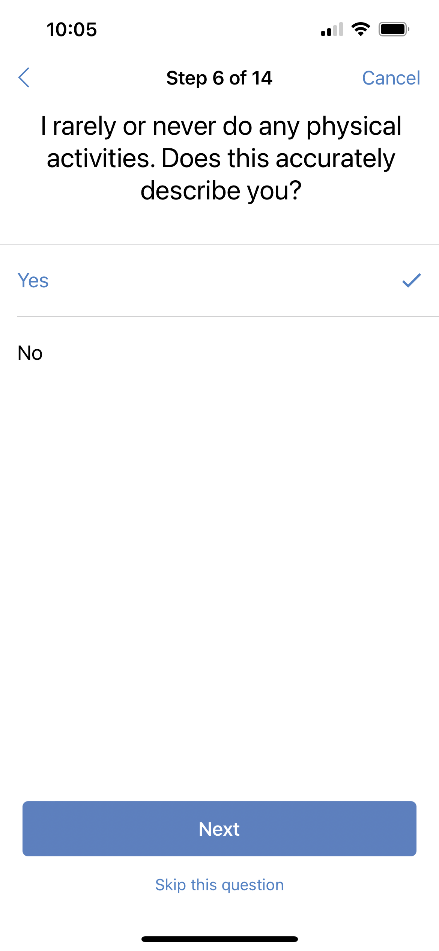
**

**
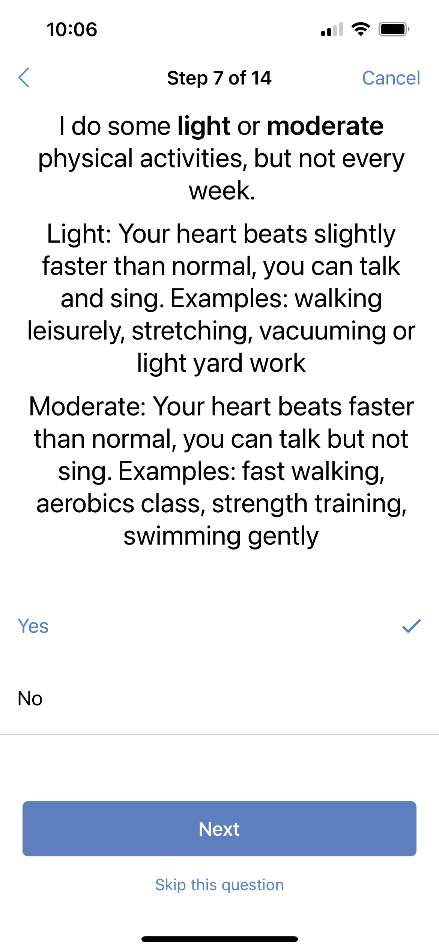

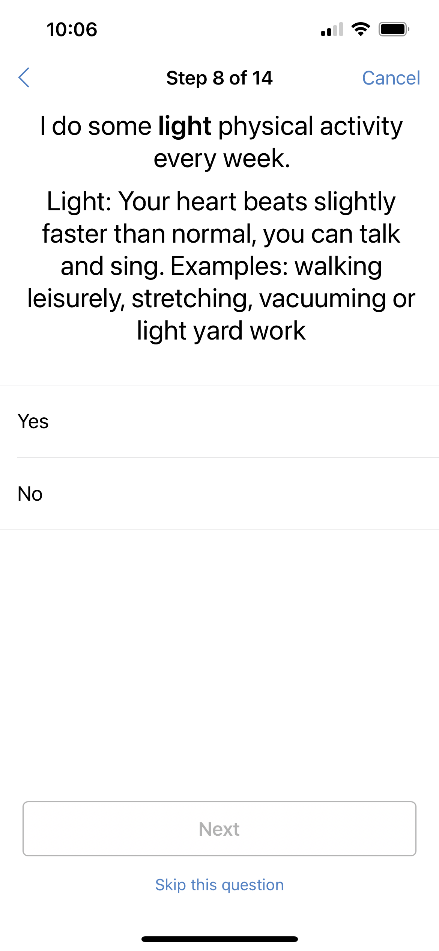

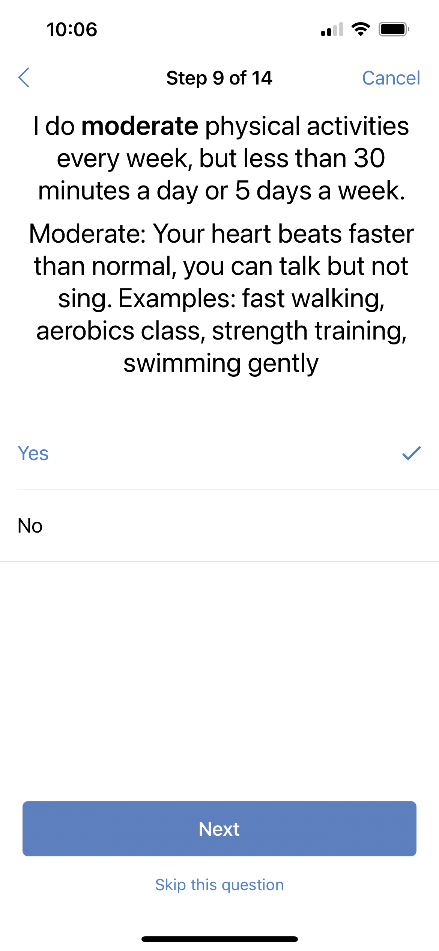

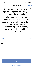

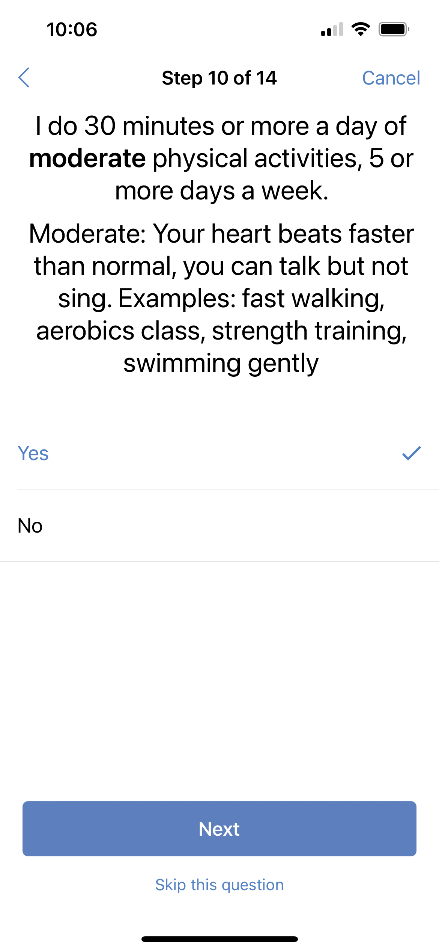

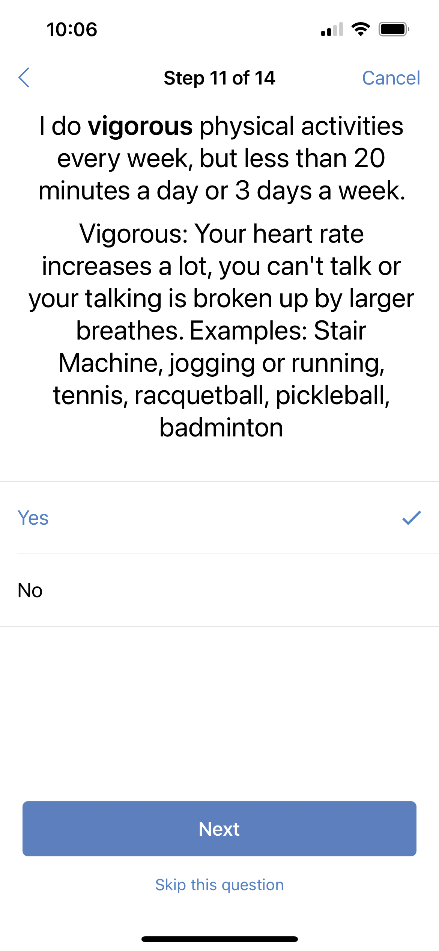

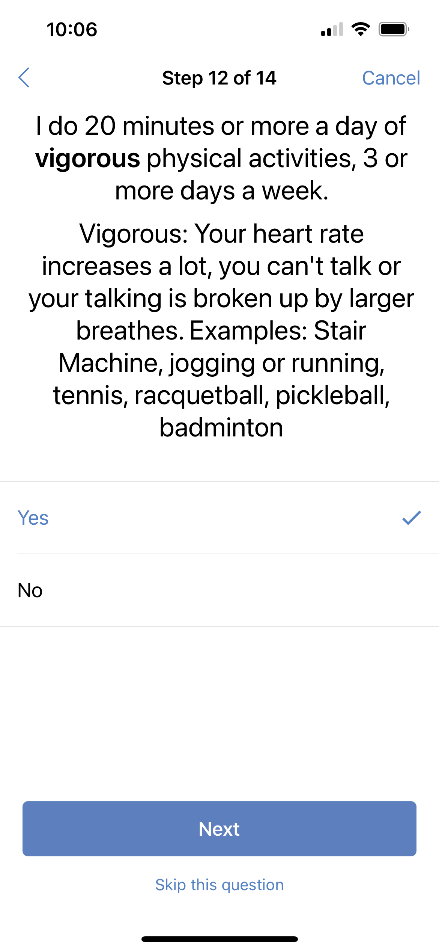

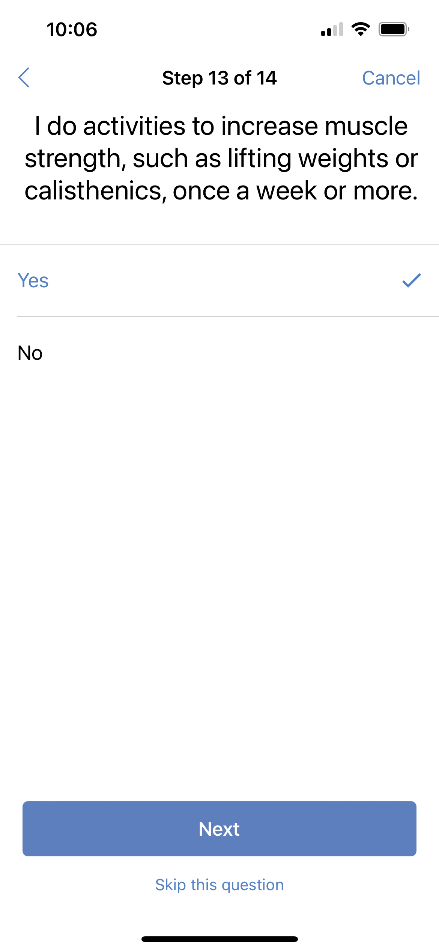

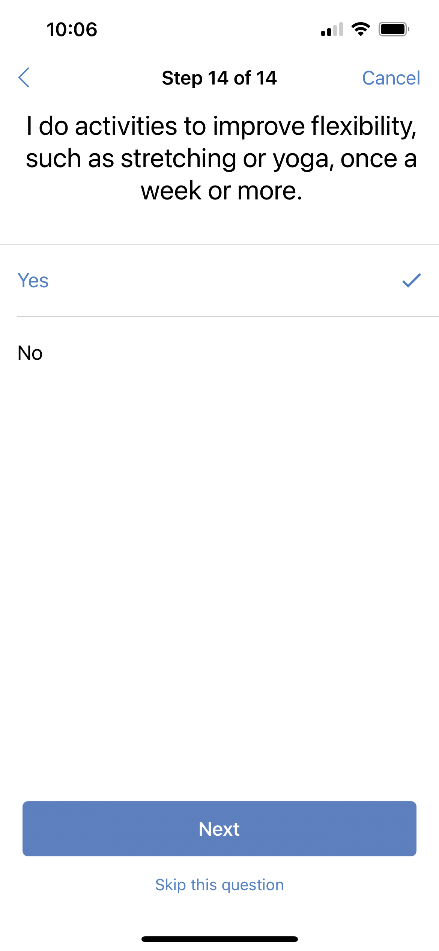

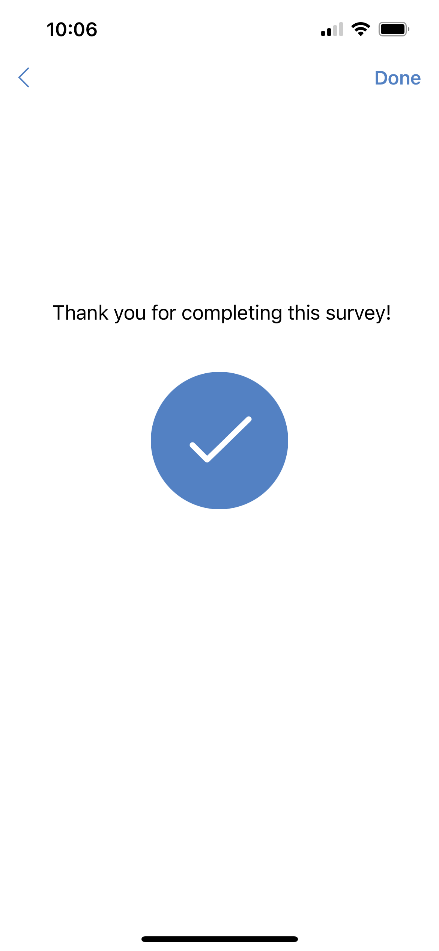
**

**Cognitive Function Survey:**

**
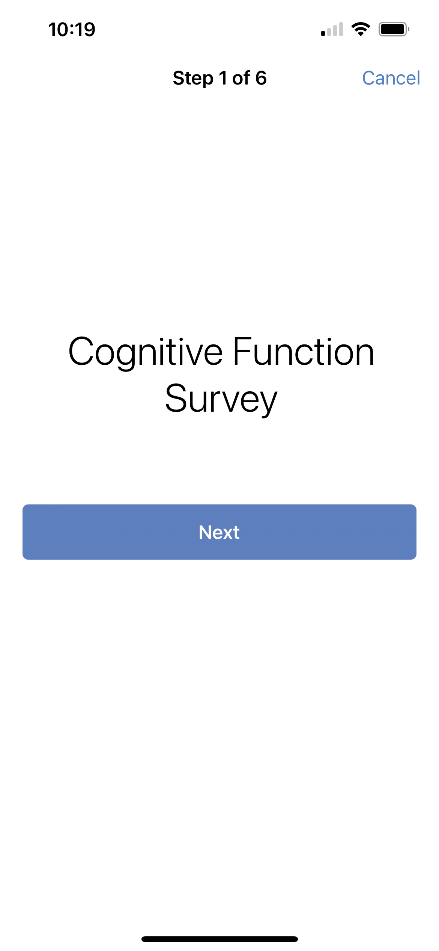

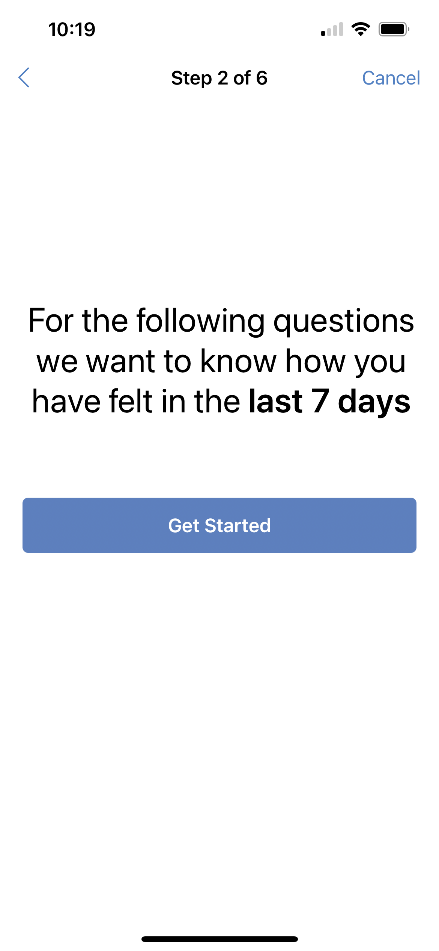

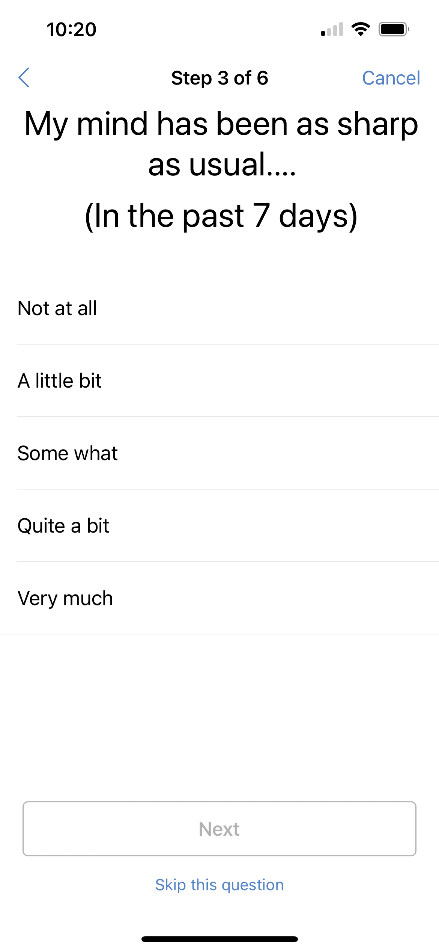

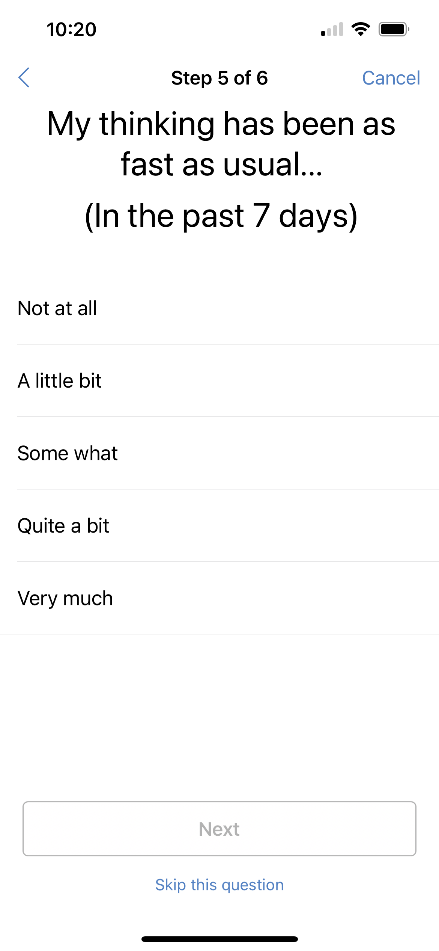

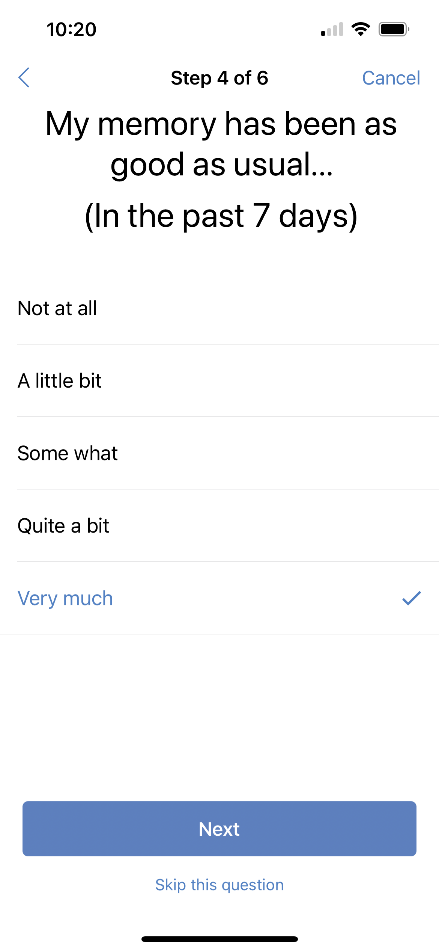

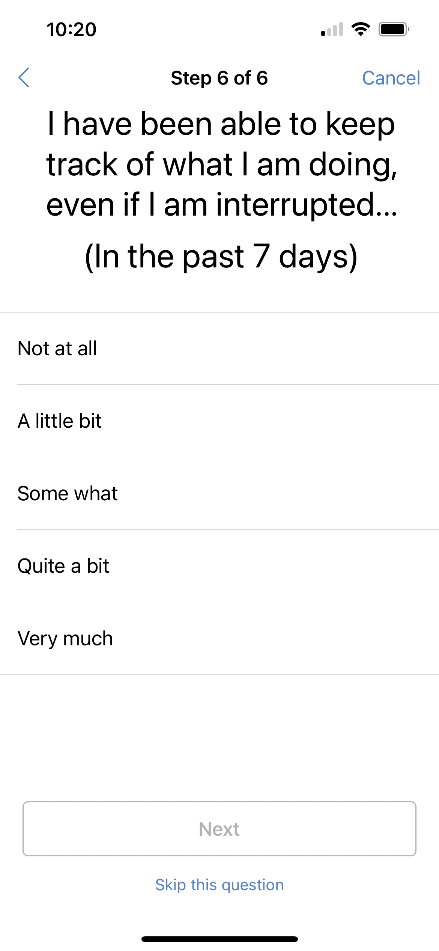

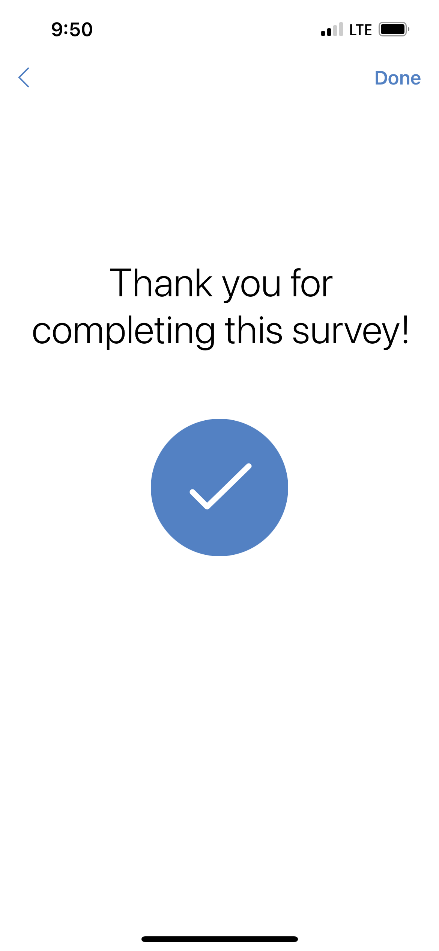
**

**Body Pain Map:**

**
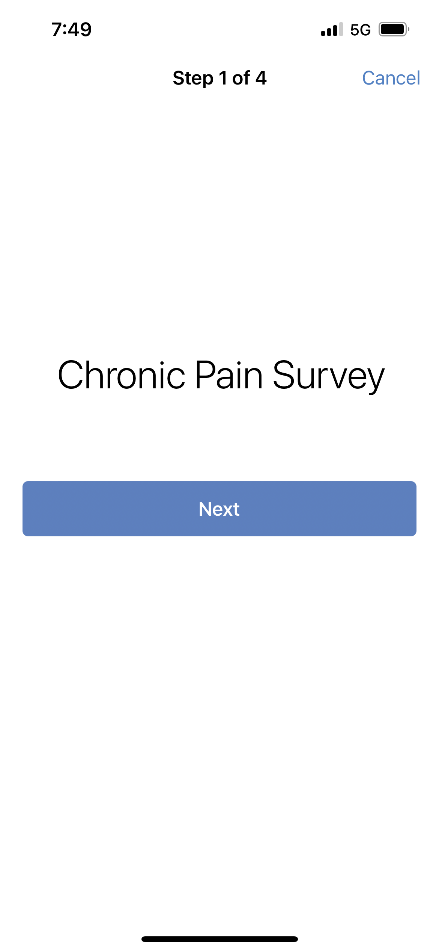

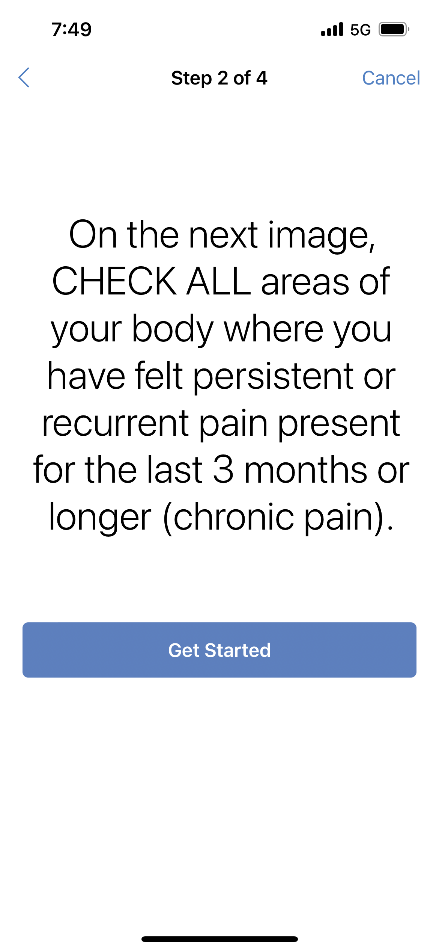

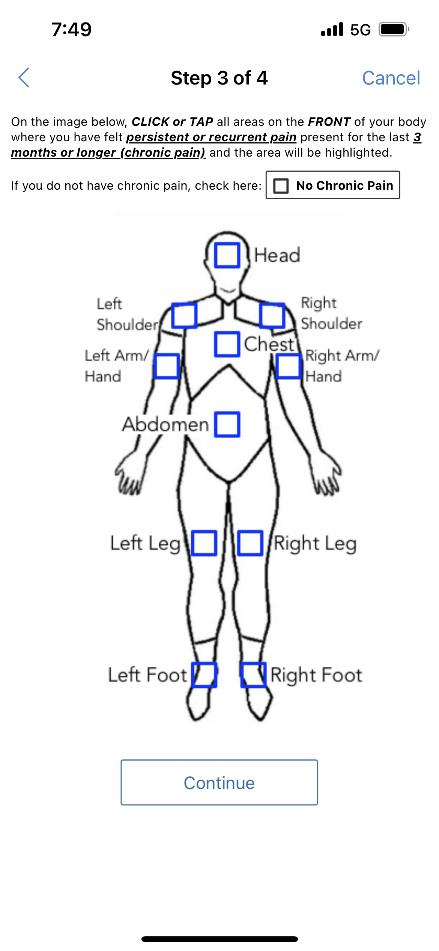

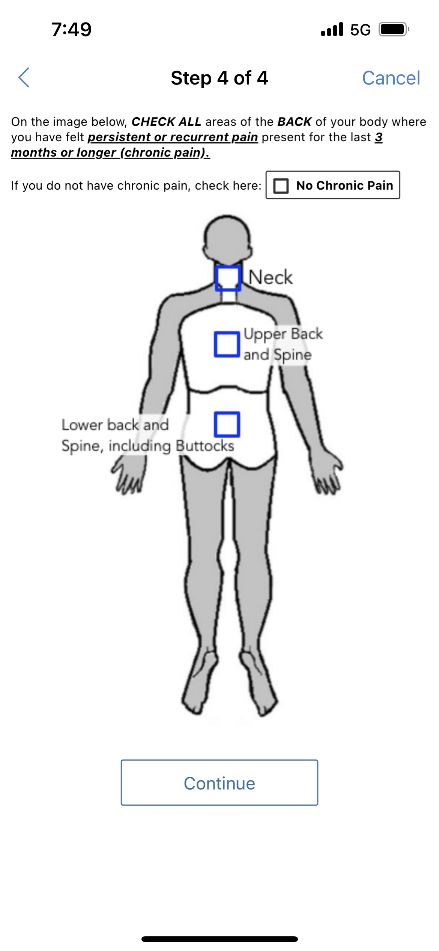

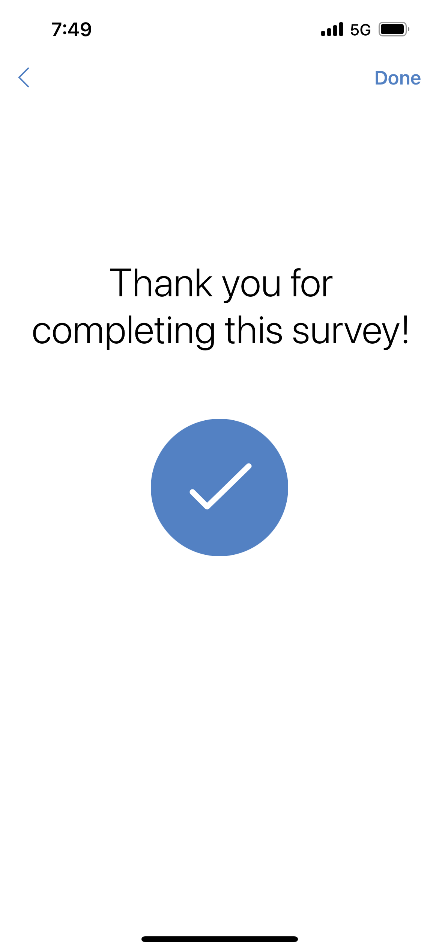
**

**Trail Making Test:**

**
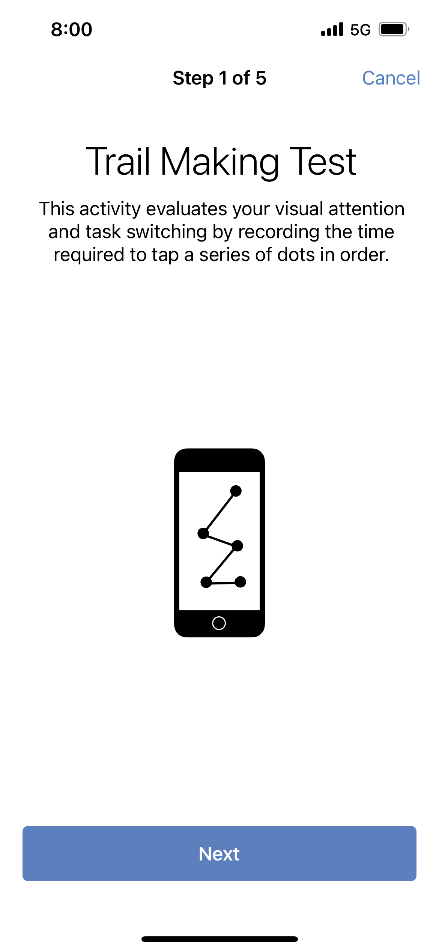

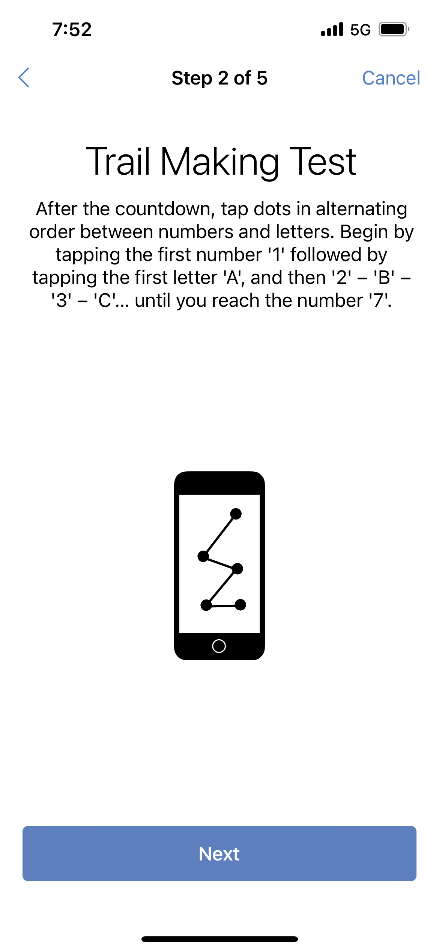

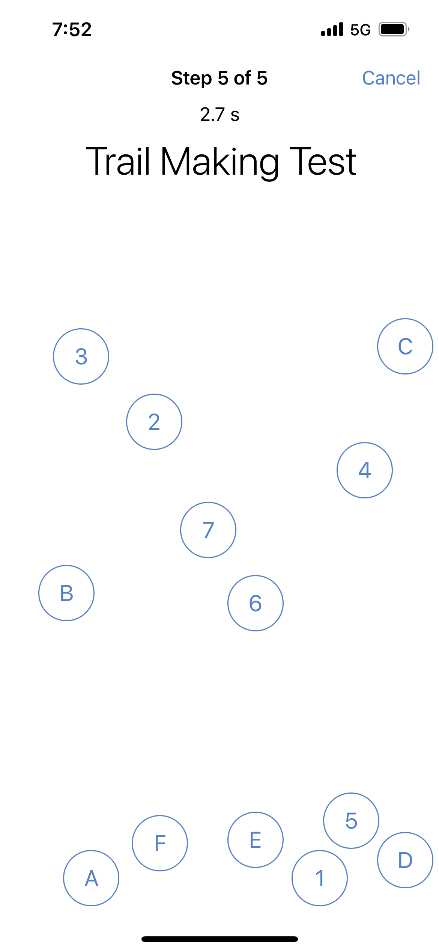

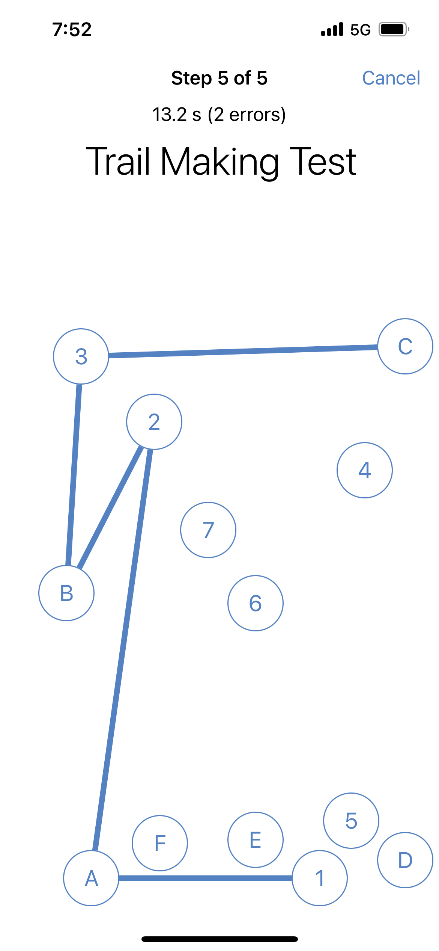

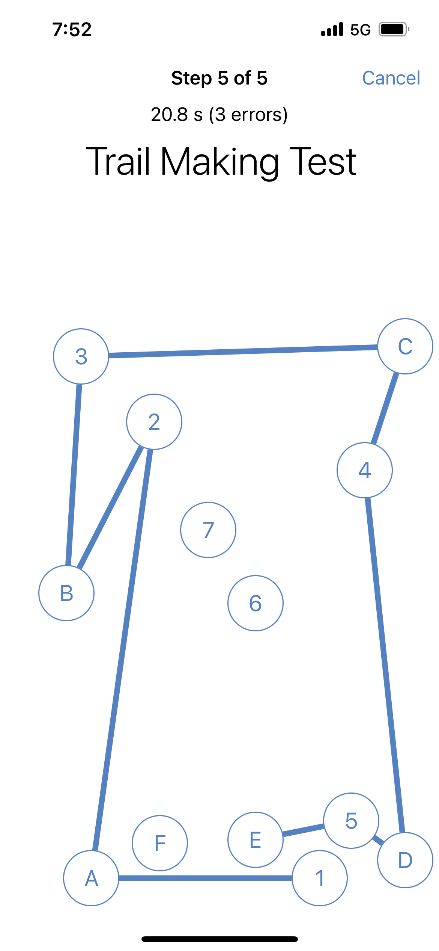

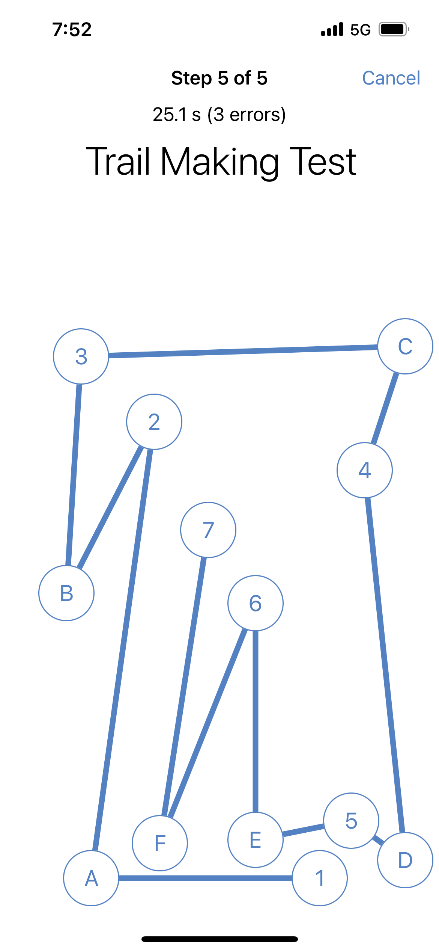
**

**
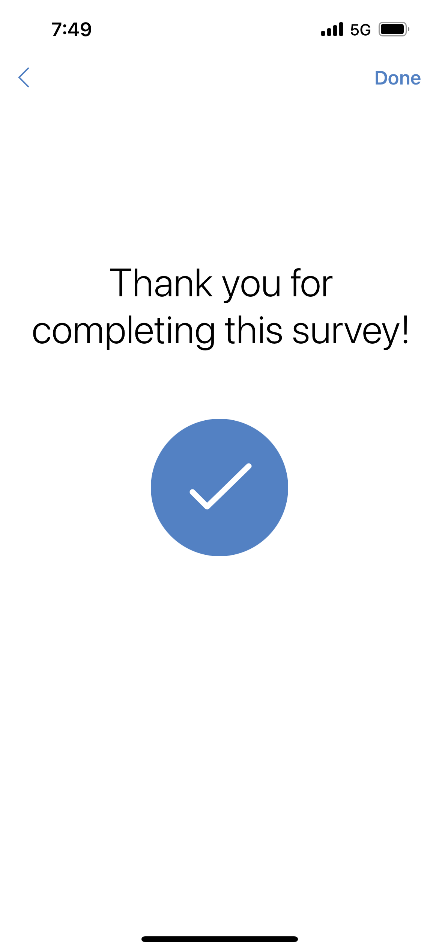
**

**STROOP:**

**
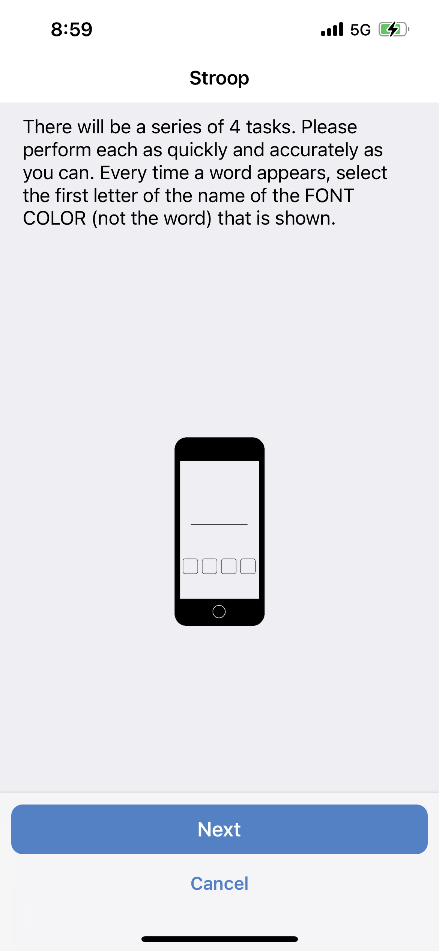

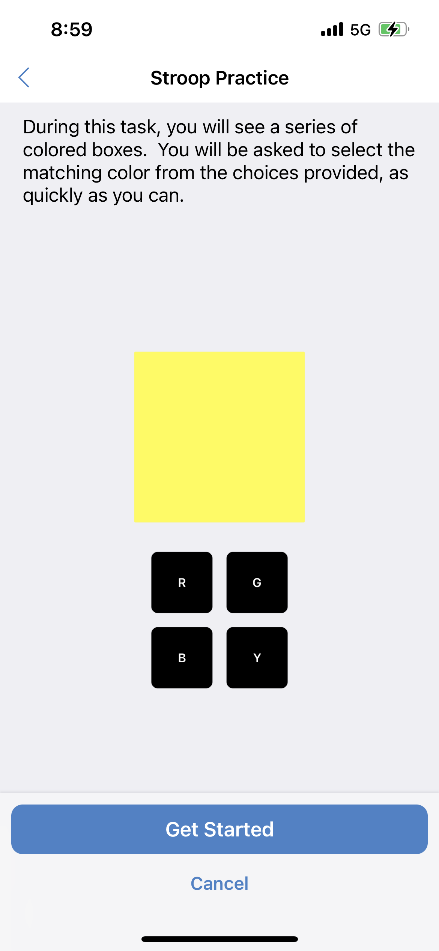

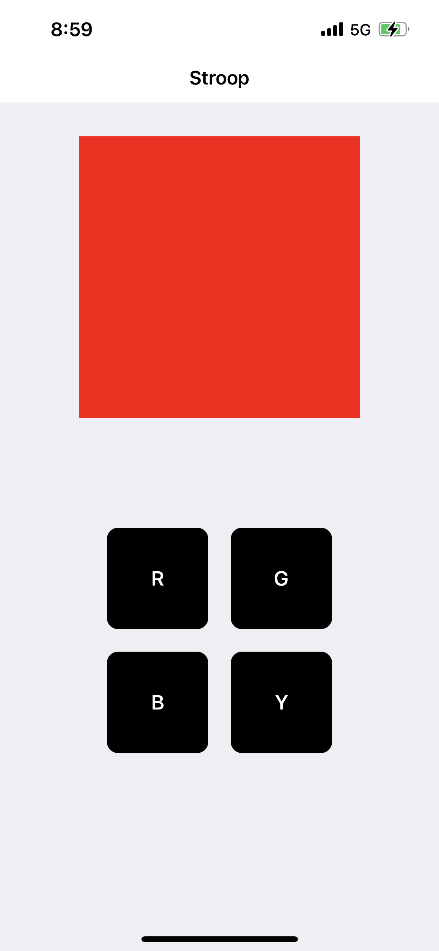

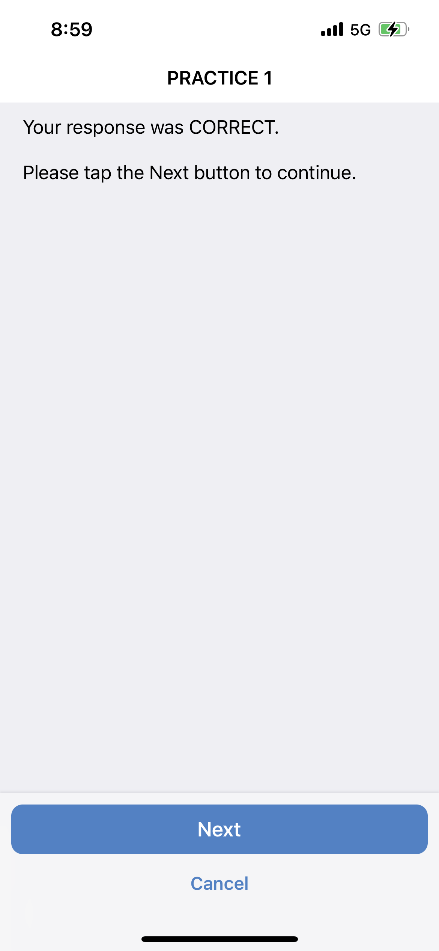

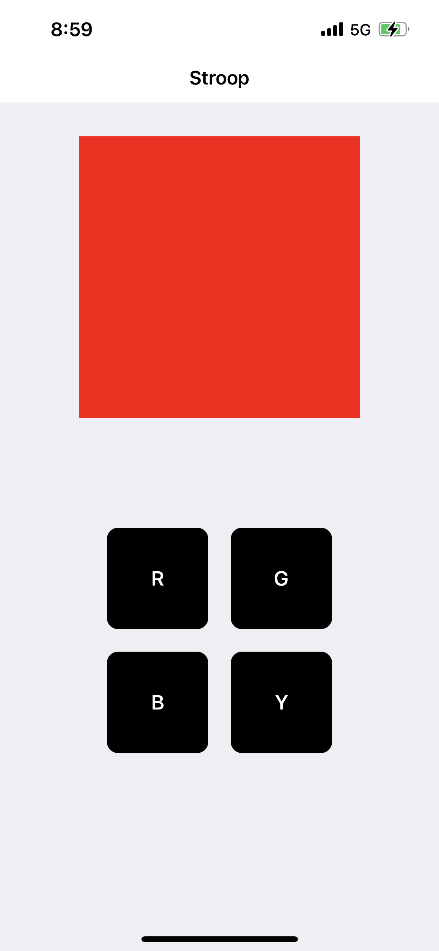

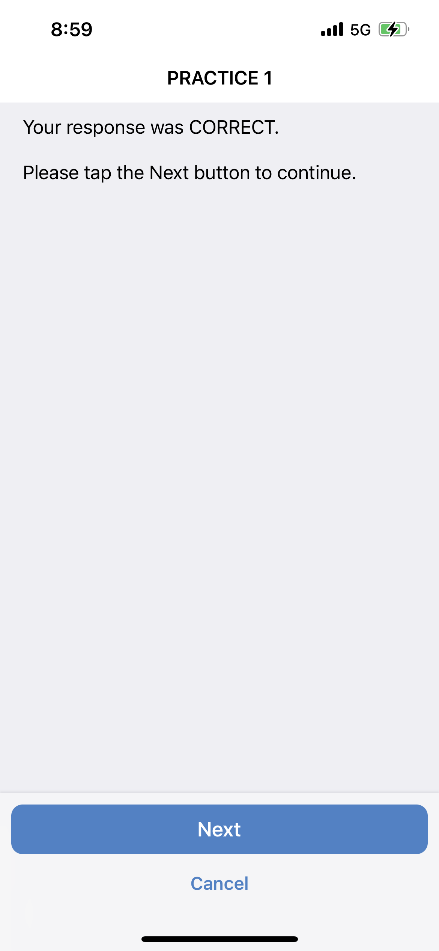

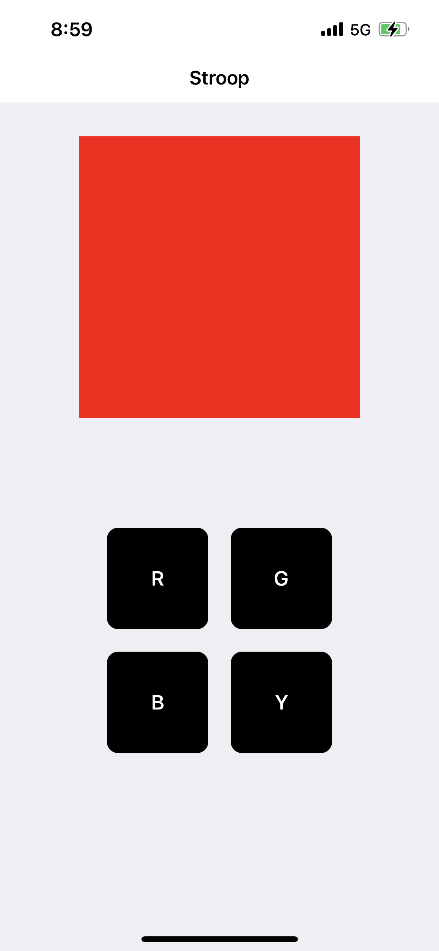

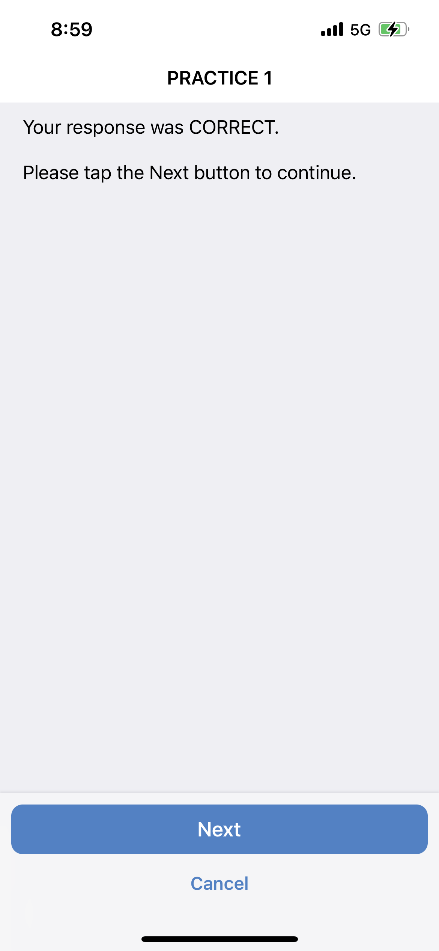

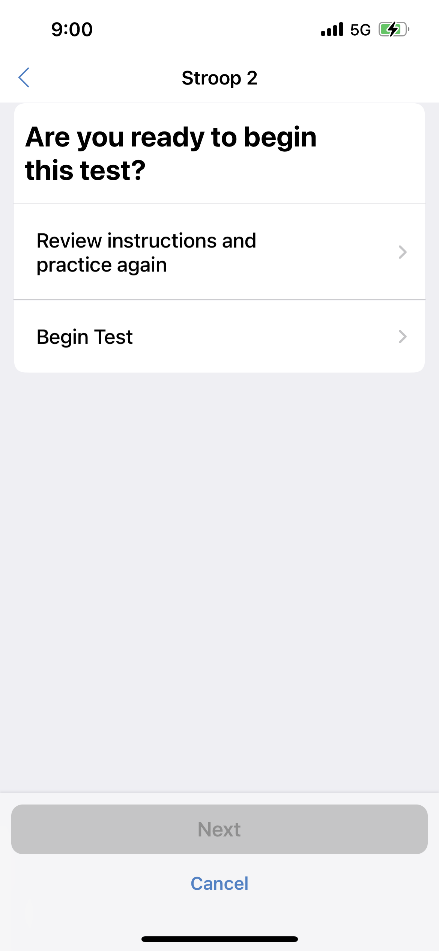

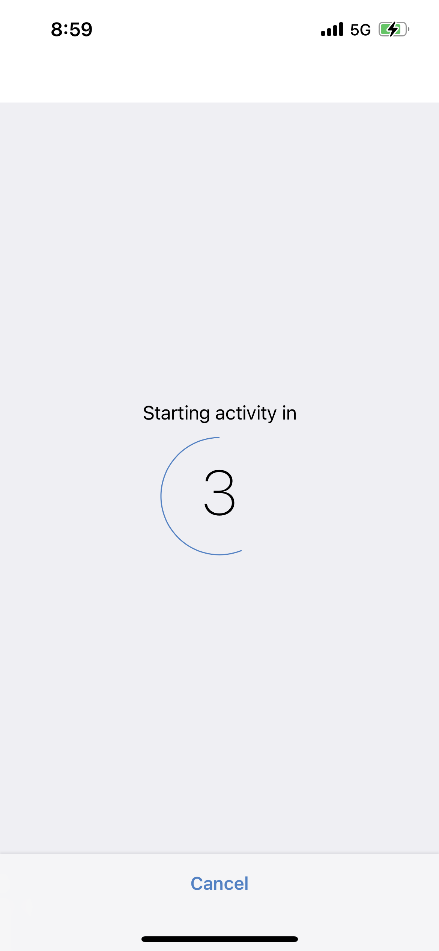

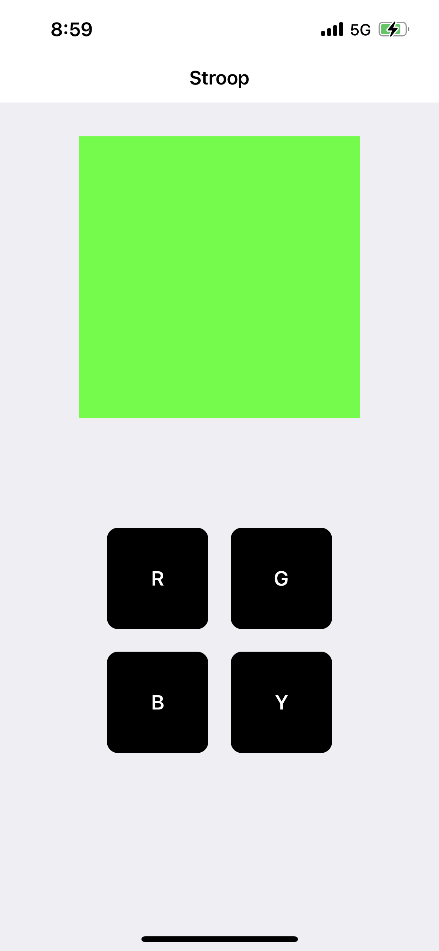

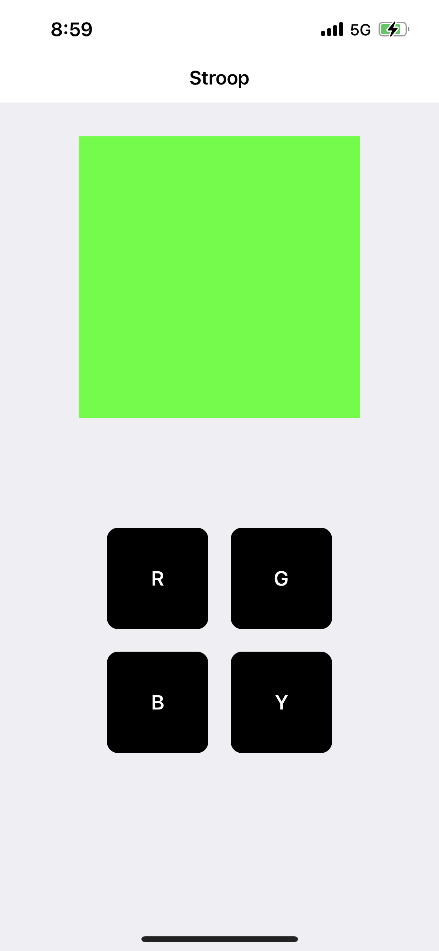

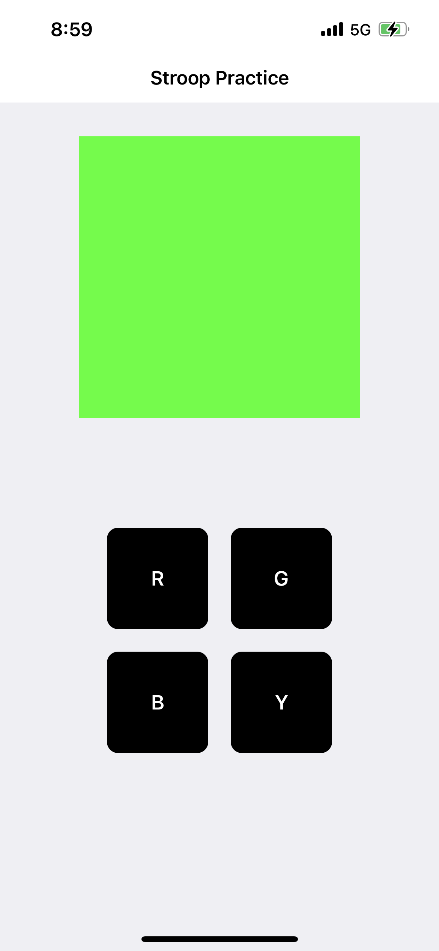

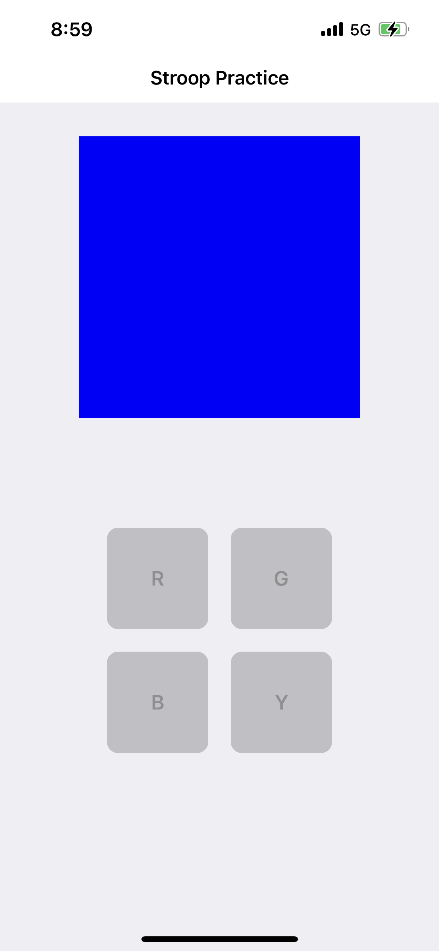

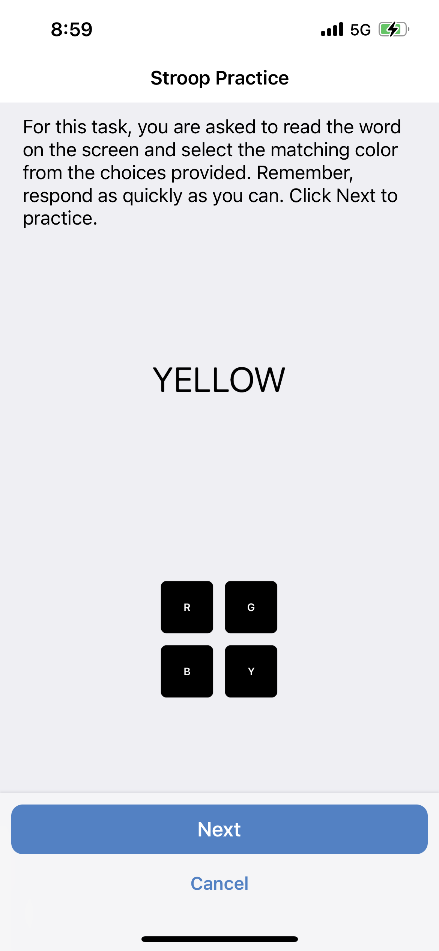

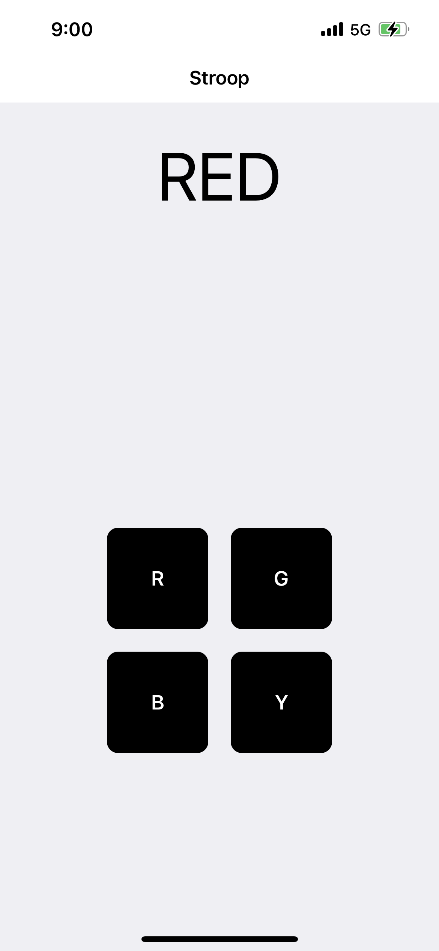

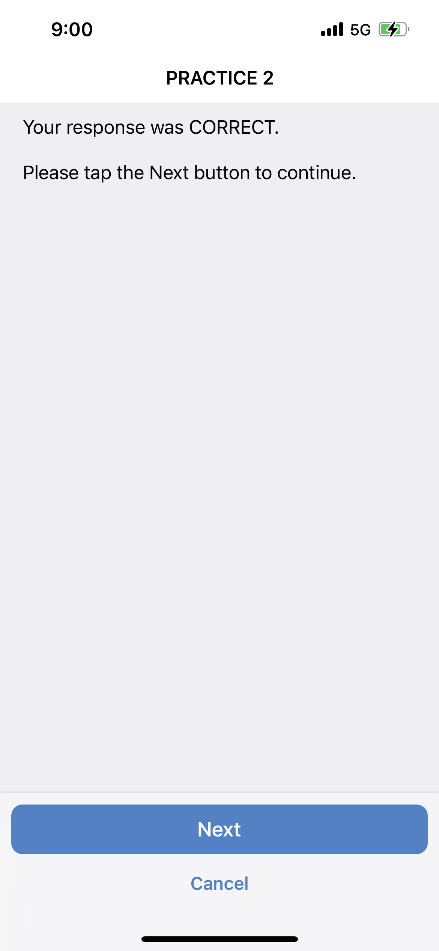

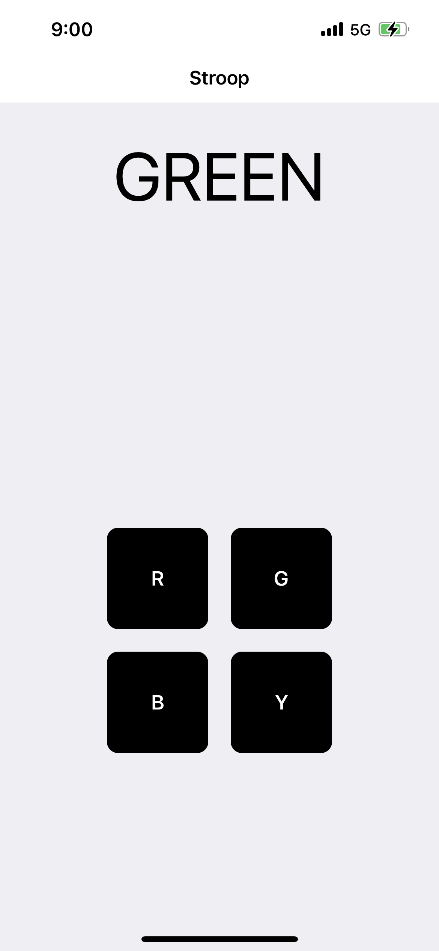

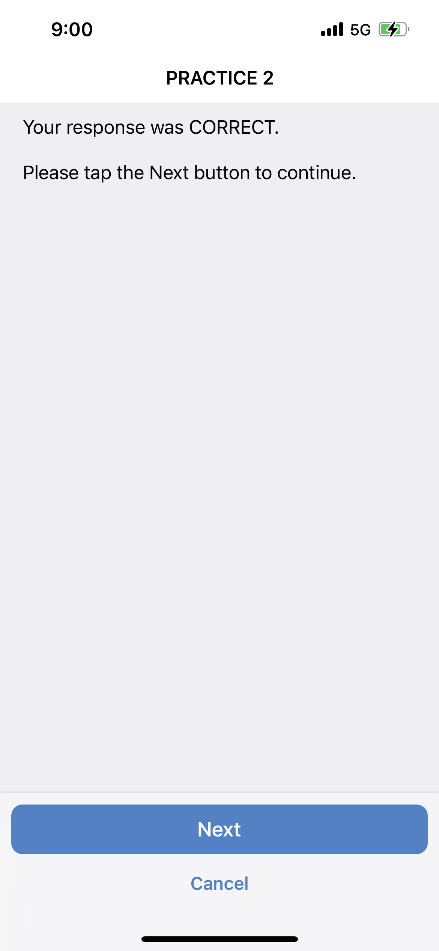

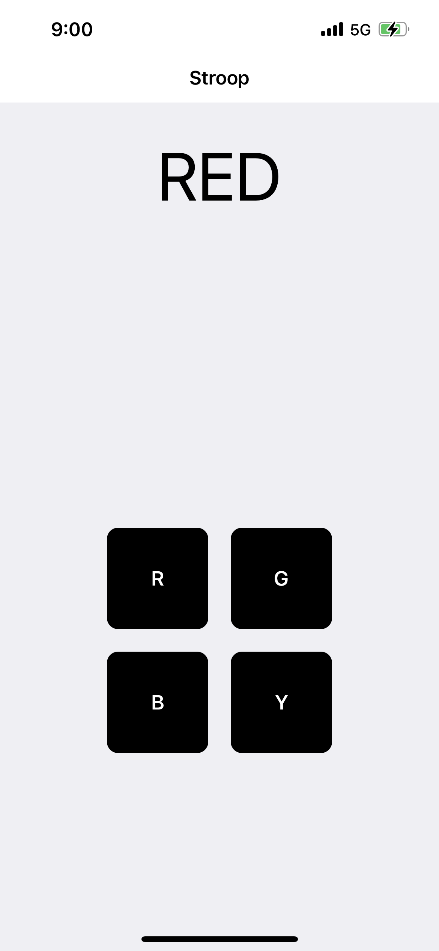

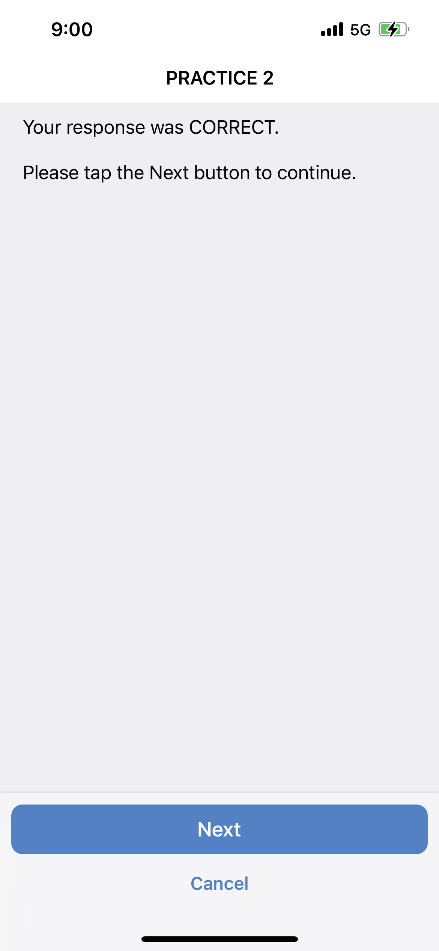

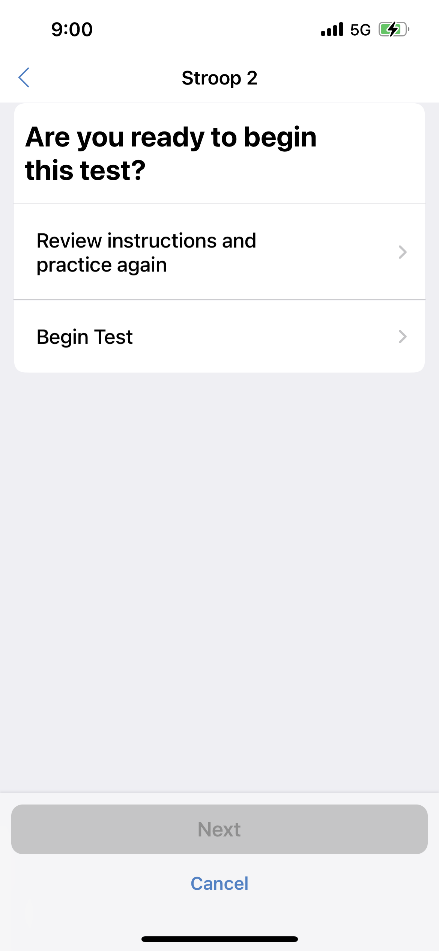

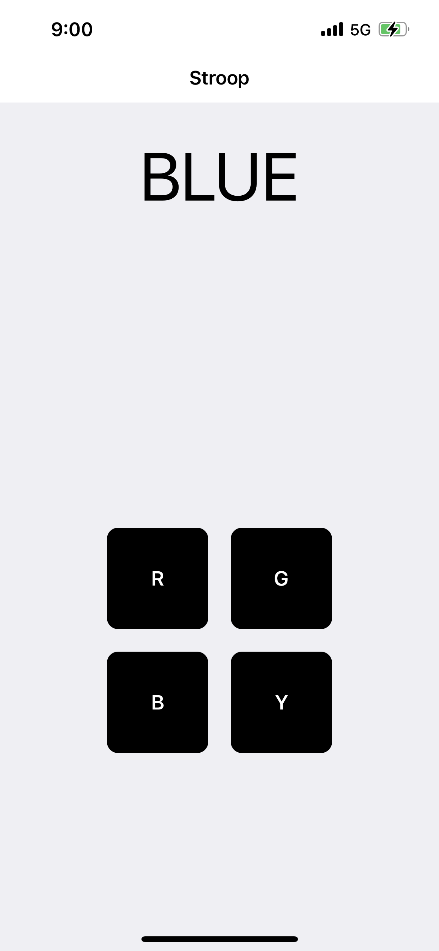

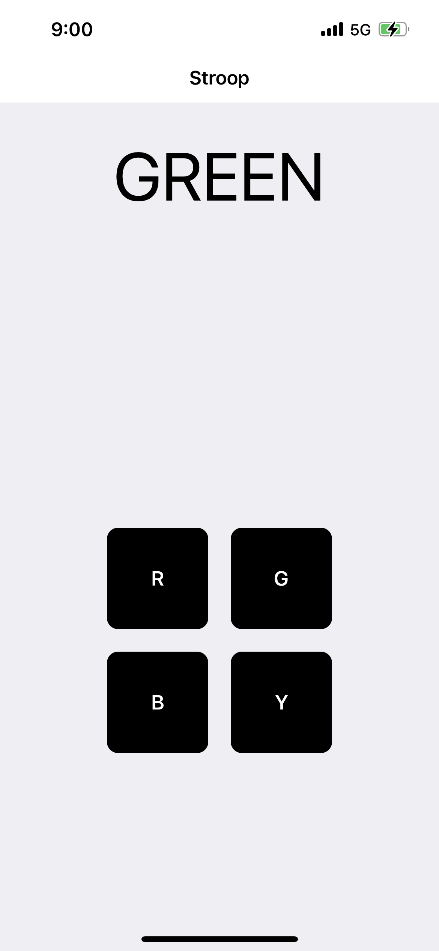

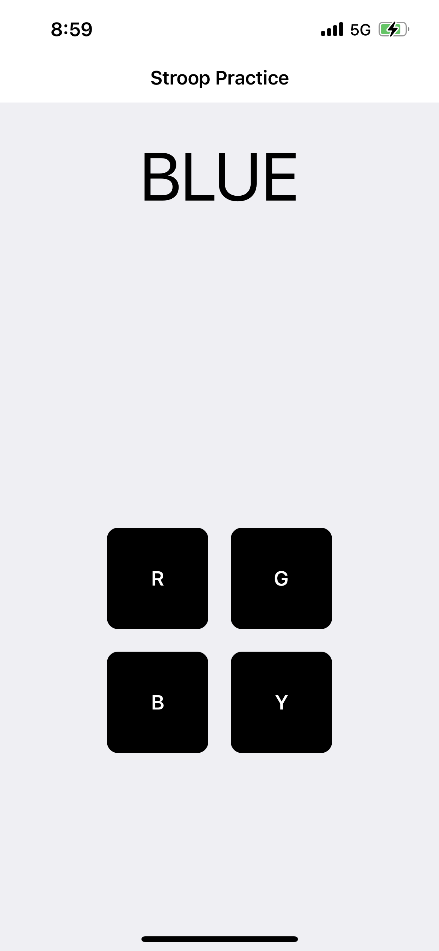

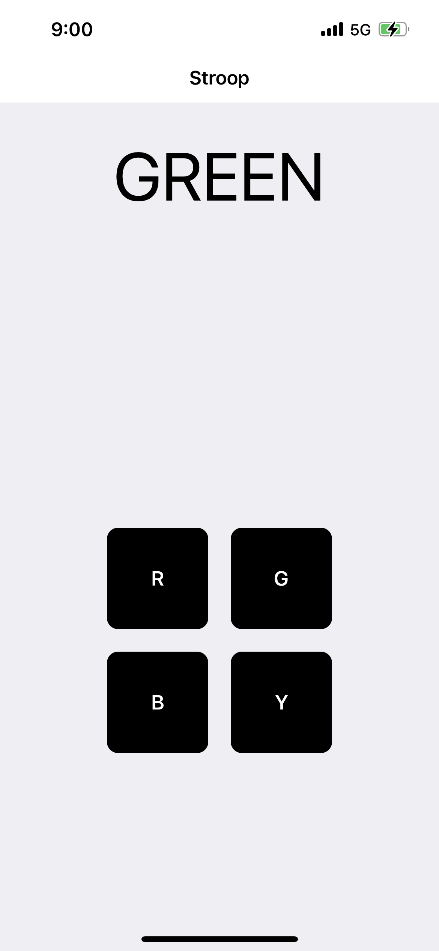

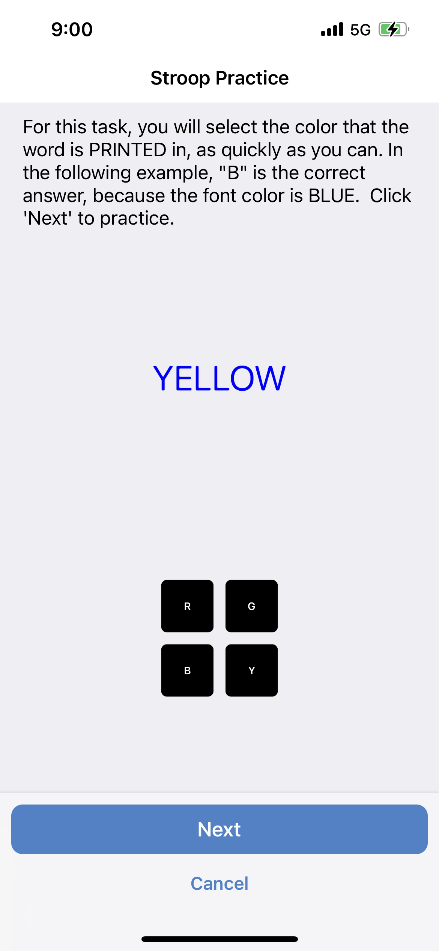

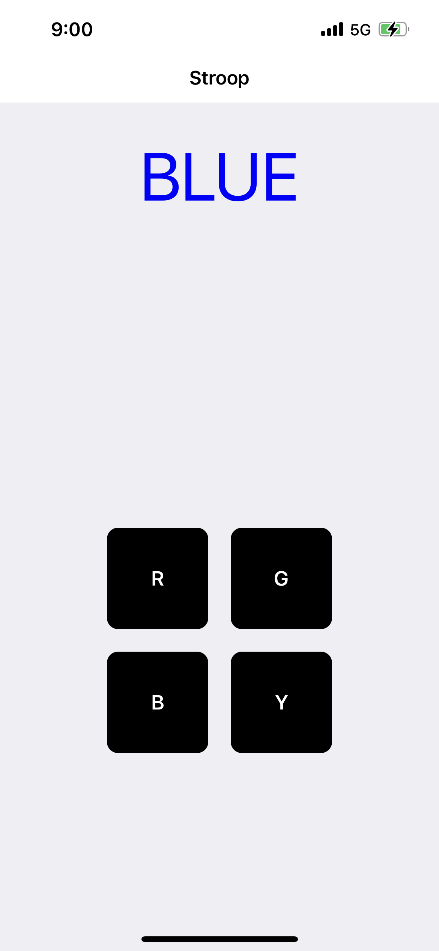

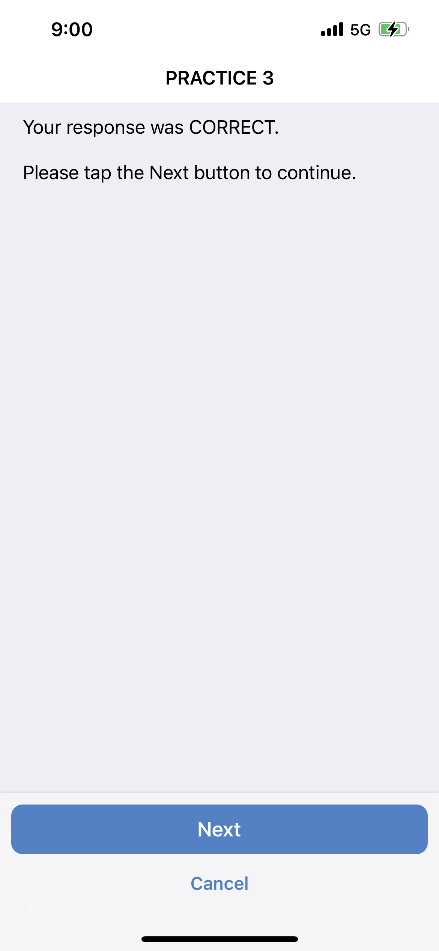

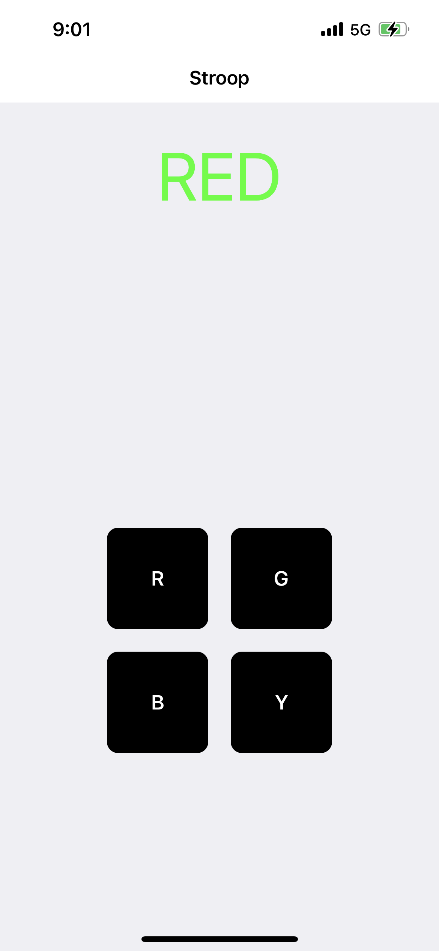

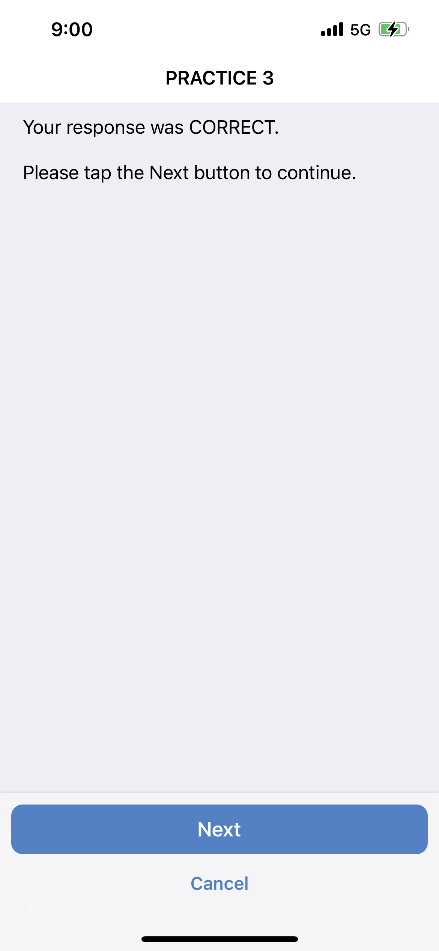

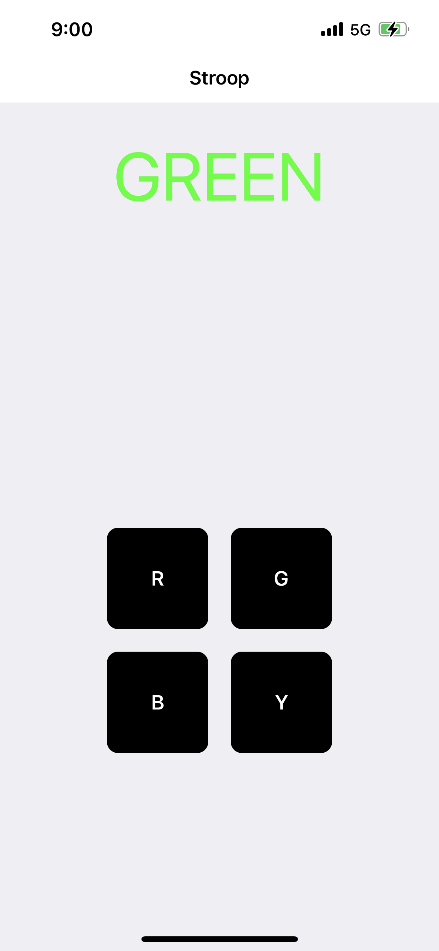

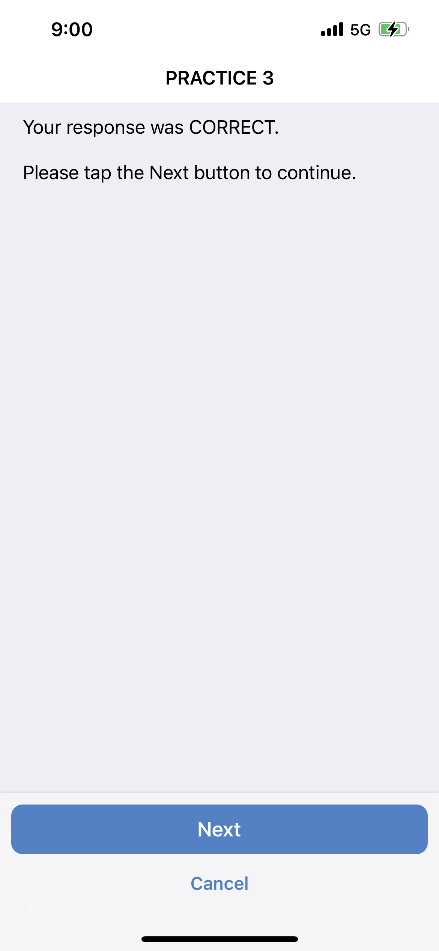

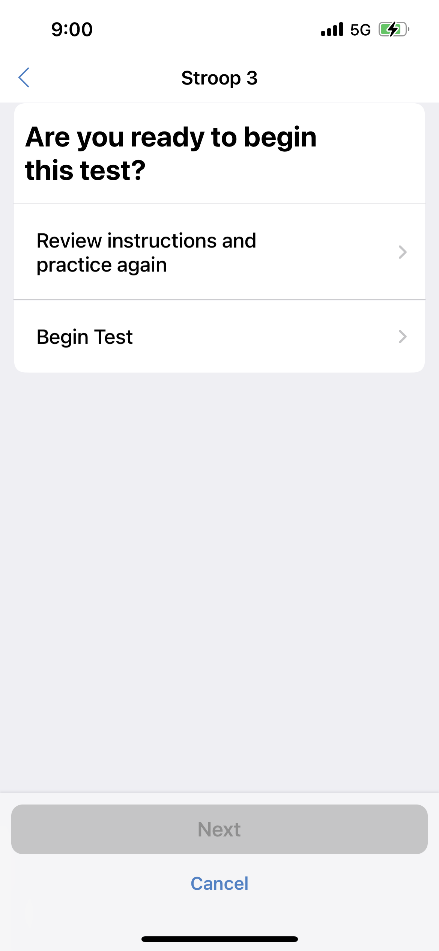

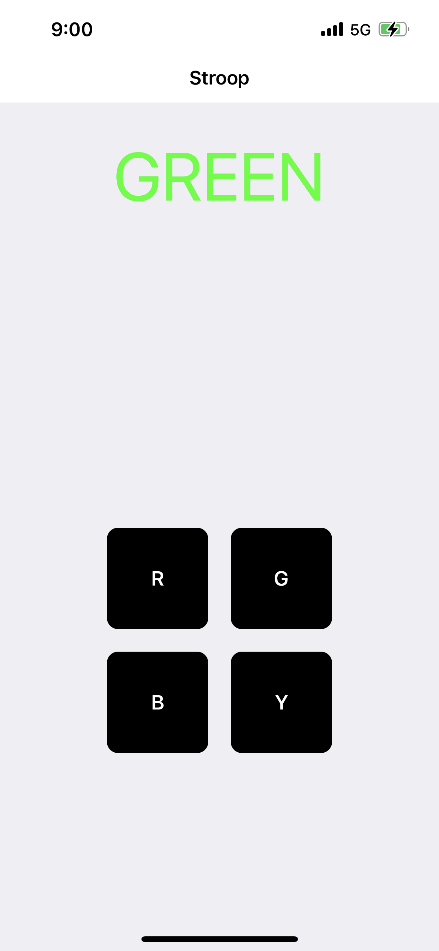

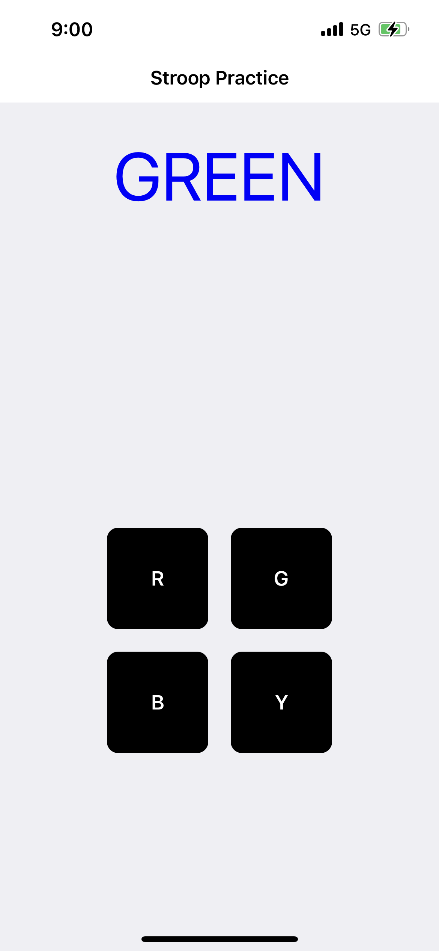

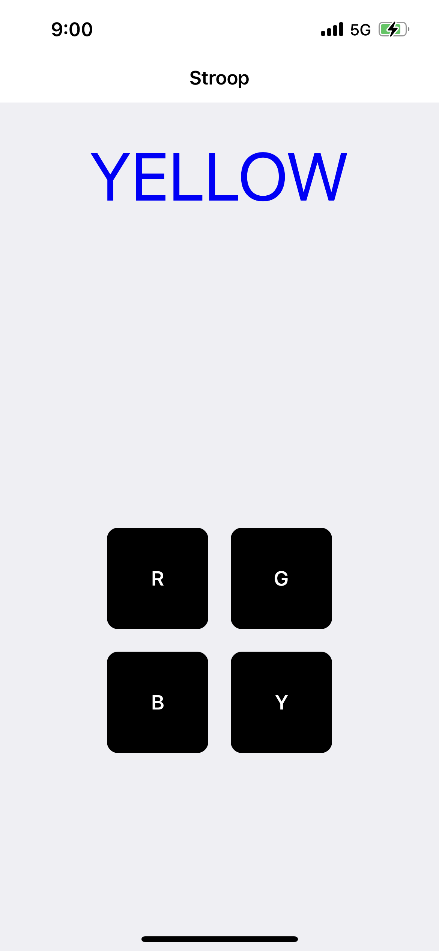

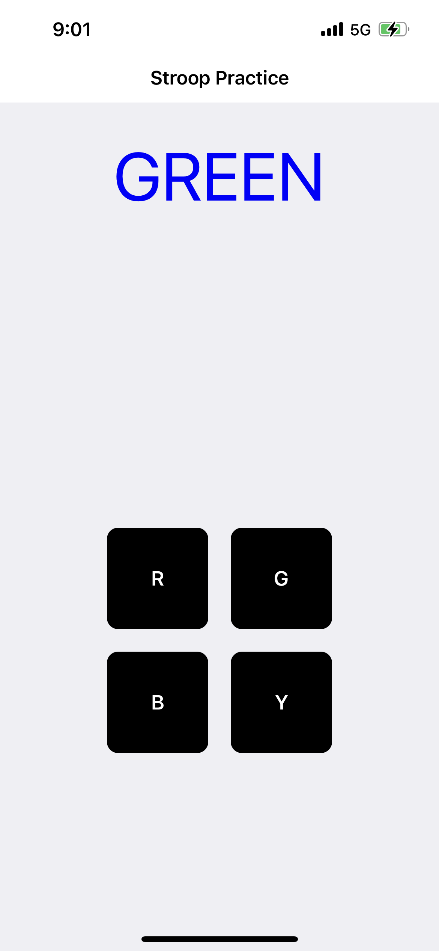

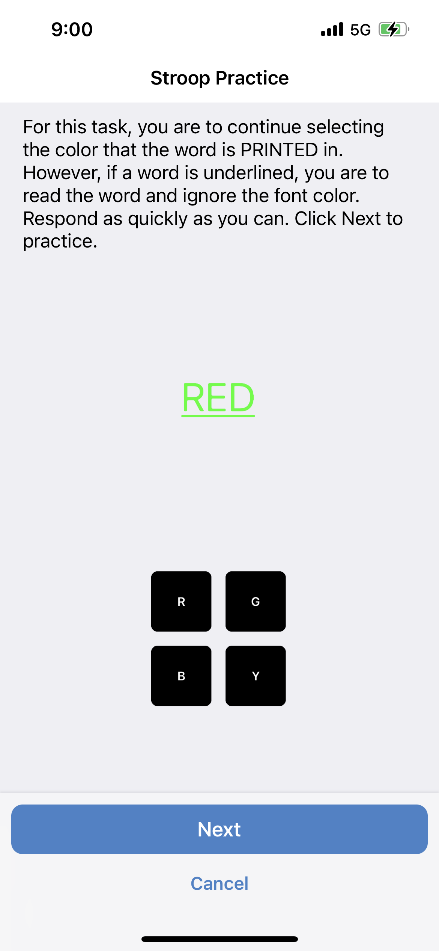

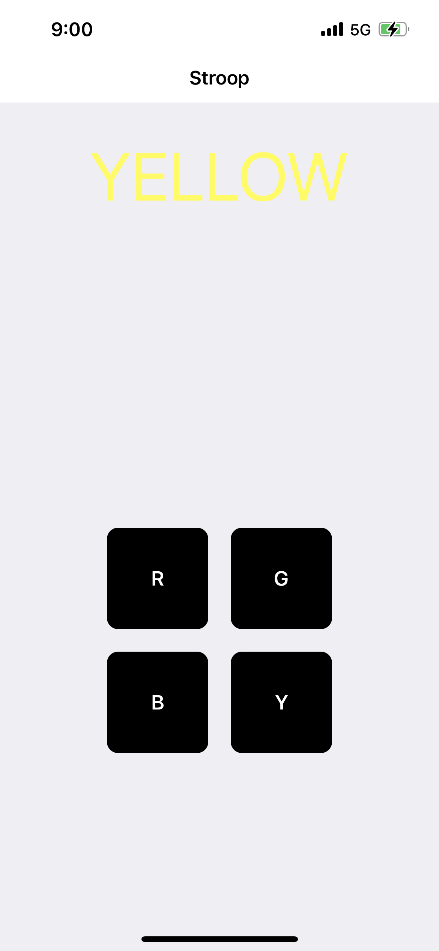
**

**Think Aloud Task Sheet- Smartphone app surveys**

Please complete the tasks below with your smartphone. While you do them, we ask that you ‘think out loud” your thoughts, what you are doing and how you are feeling so we can hear the difficulties you might be having as well as the things you like. Please say whatever comes to mind as you complete each task. The research tech was not involved in the design of the app, so please feel free to say whatever you wish, whether it’s good or bad.

Please complete the following activities using the app. Please read each step out loud so we know the step you are on as you work through the tasks.

First, find the MyDataHelps app on your phone and open the app

**Proceed to next page**

Looking at the bottom of the screen you will see the components of the app:

1. Account
2. My Projects
3. Dashboard
4. Tasks

**Task 1**. Tap on my projects and find the two studies you are enrolled in: 1) Offspring/Omni 1 and 2) Usability Study. Tap on the Usability study.

Once you are in the Usability Study, please complete the surveys in the order of your preference speaking aloud what you are doing and how you are feeling.

- Cognitive function survey
- Body Pain Map
- Mood survey
- Rapid Assessment of Physical Activity
- Falls and hospitalizations survey

If you have an iPhone please also complete the following tasks:

- Trail making
- Stroop

**Task 2**. Tap on my projects and find the two studies you are enrolled in: 1) Offspring/Omni 1 and 2) Usability Study. Tap on Offspring/Omni 1 study. Now tap on dashboard. Please describe what you see. What are your thoughts about the survey completion status area of the dashboard?

**Task 3.** Stay in the Offspring/Omni 1 project and tap on Tasks at the bottom of the screen. Under Tasks, please find the U-MARS (Mobile App Rating Survey) and complete the survey. The U-MARS will appear as the last survey in the Task list.

**Think Aloud Task Sheet- Smartphone app surveys**

Please complete the tasks below with your smartphone. While you do them, we ask that you ‘think out loud” your thoughts, what you are doing and how you are feeling so we can hear the difficulties you might be having as well as the things you like. Please say whatever comes to mind as you complete each task. The research tech was not involved in the design of the app, so please feel free to say whatever you wish, whether it’s good or bad.

Please complete the following activities using the app. Please read each step out loud so we know the step you are on as you work through the tasks.

First, find the MyDataHelps app on the phone and open the app

**This is the screen you should see.**

**Proceed to next page**

Looking at the bottom of the screen you will see the components of the app:

1. Tasks
2. Dashboard
3. Account

**Task 1**. Please tap on Tasks and complete the surveys in the order of your preference speaking aloud what you are doing and how you are feeling.

- Cognitive function survey
- Body Pain Map
- Rapid Assessment of Physical Activity
- Falls and hospitalizations survey
- Trail making
- Stroop

**Task 2**. Now tap on dashboard. Please describe what you see. What are your thoughts about the survey completion status area of the dashboard? (Screen shot of dashboard provided below)

- Next, if you had an activity tracking device, what are your thoughts about the physical activity data and goal-setting areas of the dashboard (see paper screenshot)?

**Task 3.** At the bottom of the screen, under Tasks, please find the U-MARS (Mobile App Rating Survey) and complete the survey. The U-MARS will appear as the last survey in the Task list.

Post-Procedure Interview

| **Interview Questions post think aloud procedure. The interviewer will ask questions about aspects of the tasks that were challenging to a specific participant.** |
| --- |
| 1)What are your general thoughts or impressions about the app surveys?  For example:  What did you like most about the app and why?  What did you like least about the app and why?  What, if anything was easy about it?  What, if anything was difficult about it?  What are your thoughts about the look of the app surveys, such asfont, color and layout  What are your thoughts about the way the app functioned, for example, the way it operatedand how you push the buttons.  What are your thoughts about getting from one place to the other in the app, for example, changing between screens?  What are your thoughts about the survey content? (probe—easy to understand)  The interviewer will also include more in-depth follow up questions for domain areas participant noted as challenging/difficult on the MARS (app performance, ease of use, navigation, layout, visual appeal, etc) and that the interviewer observed the participant to have difficulty with.  For example:  *I noticed you were confused using the Trail making app, what would help?* |
| 2)What positive **feelings** did you have while using the app for example, fun, excitement, interest)  What negative **feelings** did you have while using the app for example, bored, frustrated confused)  If specific surveys are mentioned:  Tell me about x that was challenging for you?  Tell me ways we could make it clearer for you?  How could we make it easier?  You mentioned being frustrated with X, can you tell me about that?  Tell me, if anything, we can do to make x clearer?  Tell me if there are other ways we can improve the surveys that you have not mentioned yet? |
| 3) To what extent do you think that people of your own age would be able to use and enjoy the app? Family, friends? |
| 4)What, if anything, do you think we could do to improve the surveys or they way you access them? |
| 5) What were your impressions of the dashboard ? How if at all will the dashboard be helpful to you? What would you change? |
| 6)How can we ensure that the experience is satisfying to people of different cultures?  What else would you like to talk about that we haven’t yet discussed  Interviewer summarizes key points and asks if there is anything the participant would like to add to the summary |
